# Juveniles and where to find them: a basin-scale habitat model for the lost years of loggerhead sea turtles in the North Atlantic

**DOI:** 10.1186/s40462-026-00640-2

**Published:** 2026-03-14

**Authors:** M. Bartolini, E. L. Hazen, H. Parra, K. A. Bjorndal, A. B. Bolten, F. Dell’Amico, T. Dellinger, R. Dietz, M. A. R. Santos, C. Sasso, N. Varo-Cruz, J. A. Bermejo Dominguez, D. Cejudo, L. F. López-Jurado, F. Vandeperre

**Affiliations:** 1 University of the Azores, Institute of Marine Sciences - OKEANOS, Rua Professor Doutor Frederico Machado 4, Horta, 9900-140 Portugal; 2 Institute of Marine Research – IMAR, Horta, Portugal; 3 Ecosystem Science Division, Southwest Fisheries Science Center, National Marine Fisheries Service, National Oceanic and Atmospheric Administration (NOAA), Monterey, CA USA; 4 Institute of Marine Sciences, University of California Santa Cruz, Monterey, CA USA; 5 Archie Carr Center for Sea Turtle Research (ACCSTR), University of Florida, Florida, USA; 6 Centre d’Etudes et de Soins pour les Tortues Marines, Aquarium La Rochelle, La Rochelle, France; 7 Centro de Investigação em Biodiversidade e Recursos Genéticos (CIBIO), InBIO Laboratório Associado, Universidade do Porto, Vairão, Portugal; 8 Estação de Biologia Marinha do Funchal, Universidade da Madeira, Funchal, Portugal; 9 BIOPOLIS Program in Genomics, Biodiversity and Land Planning, CIBIO, Vairão, Portugal; 10 Department of Ecoscience, Arctic Research Centre, Aarhus University, Roskilde, Denmark; 11 Direção Regional de Políticas Marítimas (DRPM), Horta, Portugal; 12 National Oceanic and Atmospheric Administration (NOAA), National Marine Fisheries Service, Southeast Fisheries Science Center, Miami, FL USA; 13 Cetaceans and Marine Research Institute of the Canary Islands (CEAMAR), Canary Islands, Spain; 14 Observatorio Ambiental Granadilla (OAG), Canary Islands, Spain; 15 Departamento de Biologia, Universidad de Las Palmas de Gran Canaria, Canary Islands, Spain

**Keywords:** Loggerhead sea turtle, Juvenile oceanic stage, North Atlantic Ocean, Tracking data, Habitat model, Habitat suitability

## Abstract

**Background:**

Juvenile loggerhead sea turtles represent a crucial but understudied stage of the species life cycle. Most studies have been conducted at regional scales and there remains a need for a broader scale synthesis. In the North Atlantic, loggerheads encounter numerous threats such as fishing during their transoceanic journey, so it is important to get a broad view of their distribution, movement and habitat preferences.

**Methods:**

For this study, we gathered tracking data from 124 juvenile loggerheads tagged along the Azores, Canary Islands, Madeira and in the western North Atlantic to develop a habitat model for the entire basin. We used a SSM to interpolate tracks to daily positions, simulated pseudo-absences with a correlated random walk for background data, and explored 17 environmental variables using boosted regression trees to select the best model in terms of biological realism and predictive power. The best model obtained (AUC = 0.988) was then used to predict habitat suitability for the North Atlantic for the period 1998–2022. Stranding records of loggerheads in France, UK and Ireland were used to discuss the predictions.

**Results:**

After filtering, tracks of 105 individuals, covering most of the basin, were used in the model. Our predictions of habitat suitability show the importance of the Gulf Stream and the Azorean Current. Habitat suitability is predicted to be higher in the area between 30° and 45°N all year round, while open waters below 30°N, including Cape Verde, are not predicted to be a suitable habitat. In northern and eastern areas, like the Bay of Biscay and UK and Irish waters, habitat suitability varied seasonally, with colder seasons showing lower values. Accordingly, strandings in those areas occurred mostly during winter (December-February; 45.1%) and spring (March-May; 34.1%).

**Conclusions:**

The model offers a first basin-scale prediction of the seasonal distribution of juvenile loggerheads in the North-Atlantic, which shows consistency with stranding and bycatch data in the basin. This study represents a first step towards a broader scale understanding of juveniles’ habitat preferences that can be used to quantify the magnitude and extent of the threats that they face.

**Supplementary Information:**

The online version contains supplementary material available at 10.1186/s40462-026-00640-2.

## Background

Studying sea turtles’ life cycle from land to sea has always been both challenging and ecologically important. Loggerhead sea turtles (*Caretta caretta*) have a complex life cycle which spans from neritic to oceanic habitats and covers entire oceanic basins [1]. In the ‘80s, researchers described the life cycle of loggerheads in the North Atlantic and began mapping the connectivity between the western and the eastern part of the basin, with this life history stage coined the “lost years” because of how little is known [2]. Loggerheads living in the North Atlantic hatch in the rookeries along the coasts of Central and North America and West Africa, with the largest nesting populations in Florida [3–5]. While hatchlings for western Atlantic rookeries then enter the Gulf Stream and disperse throughout the ocean basin [6, 7], dispersal from Eastern Atlantic rookeries still remains unclear and appears to be affected also by sporadic events, like storms [8]. Juveniles, with a curved carapace length (CCL) between 8.5 and 82 cm [5, 9], can be found from the central areas of the North Atlantic through the continental coasts of Portugal and North West Africa. Stranding events confirm the presence of loggerheads along the European Atlantic coast (e.g. [10–12]). Moreover, strong seasonal patterns have been highlighted in northern areas, with more strandings happening during colder seasons along the coasts of France [13], UK and Ireland [14]. Genetic studies have confirmed the connectivity between these turtles and the rookeries in the Western North Atlantic [3, 15–19], but we still lack a comprehensive understanding of how these juvenile loggerheads are distributed in space and time.

Acquiring a robust knowledge of juvenile distribution is fundamental to better understanding the life cycle of the species and protecting it. The oceanic juvenile stage of loggerhead sea turtles is a particularly sensitive life history stage in terms of conservation of the species [20] and it is important understanding, and possibly quantifying, the threats it faces. For example, juvenile oceanic loggerheads spanning from 35 to 77 cm CCL are known to interact with fisheries, mainly with longline fishing vessels targeting swordfish, sharks and tunas (e.g. [21–27]). So far, regional studies have shed some light on juvenile movements in the North Atlantic (e.g. [28–30]), while recent advances in miniaturising tags hold great promise to reveal the movements of the smallest individuals [31–33]. In contrast, the North Pacific presents a distinct scenario, where a comprehensive tagging effort included more than 200 juvenile loggerheads, both wild captured and captive reared, that have been tagged between 1997 and 2013 [34] with more deployed in recent years. This data has been fundamental to describe the oceanic habits and movement of loggerheads in the North Pacific, as they hatch in Japan and then disperse eastward, in some cases reaching the American continent [23, 34–37]. Researchers have highlighted the importance of the major currents in dispersal and movement, like the Kuroshio and the North Pacific Current, both as migratory paths and foraging areas [34, 36–38] and have found a strong connection between turtle movements and the seasonal shift of the transition zone chlorophyll front [37]. Moreover, this great amount of data has been used to add more evidence to the role of active swimming in juveniles’ movements, providing a possible explanation for the differences observed between juvenile and drifter movements [35]. These tagging and research efforts have allowed a better understanding of the threats that loggerheads are facing in the North Pacific Ocean [39, 40] and have informed management measures and tools to mitigate bycatch (e.g. [23]). Our knowledge about the oceanic habits of juveniles in the North Atlantic to date suffers from a high level of fragmentation in tagging effort and synthesis. Therefore, it is critical to aggregate tag data from across the North Atlantic to better understand juvenile movements and habitat preferences at the basin scale.

Habitat modelling is a powerful and well-studied tool to integrate data from different sources and understand how species interact with their environment across multiple scales. Many techniques have been developed and widely used [41–43], varying across parametric (e.g. generalised linear mixed models, GLMMs), semi-parametric (e.g. generalised addictive mixed models, GAMMs) and non-parametric (e.g. boosted regression trees, BRTs) modelling approaches. Each technique has particular strengths and weaknesses, but they ultimately share a common goal: quantifying a relationship between habitat variables and the species distribution for inference and prediction [41]. For example, BRTs are particularly useful when it comes to deal with a great amount of data, thereby enabling the use of the full dataset without omissions. Moreover, because of their non-parametric nature, BRTs can cope with collinearity between explanatory variables, making it possible to test the influence of several variables that may be correlated [44, 45]. Because of these reasons and the fact that they have good predictive power, BRTs represent an interesting tool when the research goal is to make predictions, however, they may be less effective when comparing their explanatory power with other modelling techniques [44, 46]. Most of the commonly used modelling methods compare the environment where the species is present (presence data) with the environment where the species is absent or assumed to be absent (pseudo-absence / background data, generated when it is impossible to obtain true absence data, like in the marine environment). Habitat models can be used with presence-only data, like tracking data, to model the species’ preferred habitat and make predictions of their distribution, both in terrestrial and marine environments [47–49]. Habitat models can have many applications, such as spatial management of different types of anthropogenic pressures, like fisheries or shipping (e.g. [23, 50, 51]), the creation and/or evaluation of existing protected areas (e.g. [50, 52–54]). and the evaluation of possible effects of climate change on a species distribution and movements (e.g. [55]).

This study presents the first habitat model for juvenile loggerhead sea turtles covering the entire North Atlantic. The model is based on tracking data collected across multiple research teams and study sites throughout the basin. The main goals of this study are merging the information available about juveniles in the North Atlantic, identifying areas with higher habitat suitability, and highlighting seasonal patterns of habitat quality. This habitat model then set the stage for threat assessments [56], management tools (e.g. [23]), and prediction of future states linked to climate change (e.g. [55]).

## Materials and methods

### Tracking data

**Fig. 1:**
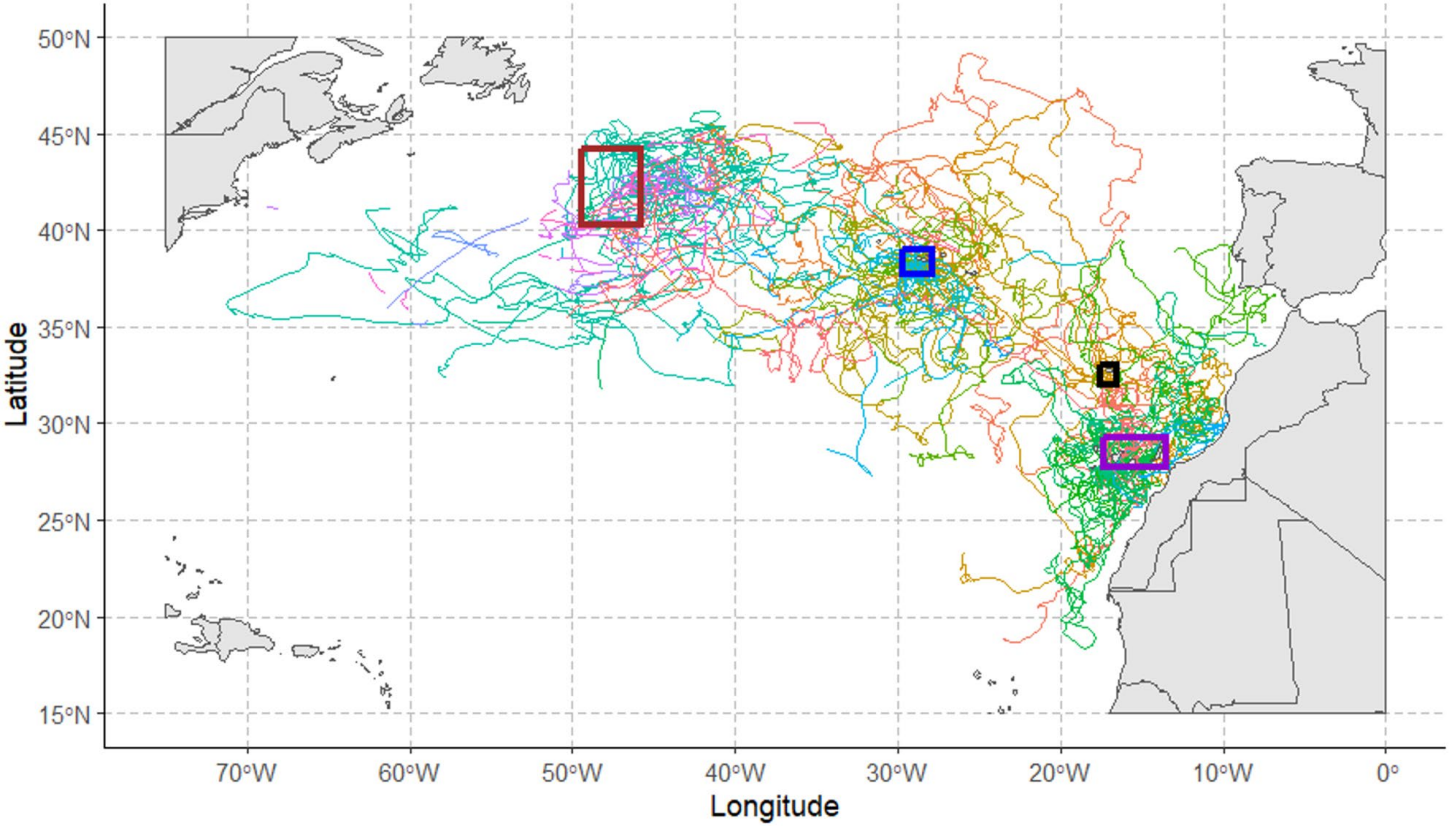
Tracking data. Segments used as presence data. The shown segments have been obtained after filtering and elaborating raw data collected by Argos and geolocators tags (see Materials and Methods for details). Each different colour represents a different segment. Rectangles represent the tagging and release areas: brown for Central North Atlantic, blue for the Azores, black for Madeira and violet for the Canary Islands

The dataset collected from the participating research teams includes tracking data from 124 juvenile loggerhead sea turtles. Individuals were captured in 4 areas of the North Atlantic basin (Fig. 1) and were tagged with Argos platform transmitter terminals (PTTs) or with geolocators. Body size spanned from 32.4 to 74.0 cm CCL, with an average of 51.8 ± 8.6 cm (mean ± sd, Table 1-S1) and no significant difference in size distribution between tagging areas (Fig. S1). The observed CCL values are consistent with the juvenile stage of the species [5, 6, 57], making this dataset representative of the North Atlantic population of juvenile loggerhead. Tracking duration varied between 11 and 951 days, with an average of 243 ± 162 days (mean ± sd, Table 1-S1). Details about tag design, duty cycles and individuals can be found in Table 1 and Table S1.

**Table 1:** Summary of individuals and tags information. Information about turtles tagged by area. Details about each tagged individual can be found in Table S1

Tagging area	Number of individuals	CCL [cm] (mean ± sd)	Capture condition	Tag type	Tracking days (mean ± sd)
Azores	42	52.7 ± 8.6	Wild (35), light hooked (7)	Argos PTTs	191 ± 122
Canary Islands	24	51.1 ± 8.6	Wild	Argos PTTs	287 ± 230
Central North Atlantic	48	51.9 ± 10.3	Wild	Geolocators	270 ± 155
Madeira	10	49.5 ± 7.2	Wild	Argos PTTs	226 ± 105
All areas	124	51.8 ± 8.6	Wild (117), light hooked (7)	Argos PTTs (76), geolocators (48)	243 ± 162

#### Azores

Forty-two juvenile loggerhead sea turtles were tagged between 1994 and 2005. Individuals were wild captured by hand (35, Table S1) or caught as bycatch in the surface longline fishery (7, Table S1). The latter only included turtles with light injuries that didn’t affect their behaviour [28, 58, 59]. Body size varied from 35.0 to 74.0 cm CCL, with an average value of 52.7 ± 8.6 cm (mean ± sd, Table 1-S1). All the individuals were tagged with PTTs produced by Wildlife Computers, USA (10, Table S1), or Telonics, USA (32, Table S1). Individuals were released in open waters, close to the islands of Faial and Pico (Fig. 1). The tracking period lasted from a minimum of 11 days to a maximum of 705 days, with an average of 191 ± 122 days (mean ± sd, Table 1-S1).

#### Canary Islands

Twenty-four juvenile loggerhead sea turtles were tagged between 1999 and 2009. All individuals were wild captured and were visibly healthy [30]. Body size varied from 36.5 to 58.0 cm CCL, with an average value of 51.1 ± 5.1 cm (mean ± sd, Table 1-S1). All the individuals were tagged with PTTs produced by Telonics, USA (5, Table S1), or Sirtrack, New Zealand (19, Table S1). Individuals were released from shores of the islands of Tenerife, Gran Canaria and Lanzarote (Fig. 1). The tracking period lasted from a minimum of 24 days to a maximum of 951 days, with an average of 287 ± 230 days (mean ± sd, Table 1-S1).

#### Central North Atlantic

Forty-eight juvenile loggerhead sea turtles were tagged between 2009 and 2012. All individuals were dip-netted from the surface by commercial fishermen and tagged following [60]. Body size varied from 32.4 to 73.9 cm CCL, with an average value of 51.9 ± 10.3 cm (mean ± sd, Table 1-S1). All the individuals were tagged with geolocators produced by Wildlife Computers, USA (Table S1). Geolocators were programmed to record daily light levels and sea surface temperature (SST) and to transmit this data through Argos once a month. Individuals were released in the Central North Atlantic (Fig. 1). The tracking period lasted from a minimum of 20 days to a maximum of 538 days, with an average of 270 ± 155 days (mean ± sd, Table 1-S1).

#### Madeira

Ten juvenile loggerhead sea turtles were tagged in 1998. All individuals were wild captured by hand [29]. Body size varied from 37.9 to 63.1 cm CCL, with an average value of 49.5 ± 7.2 cm (mean ± sd, Table 1-S1). All the individuals were tagged with PTTs produced by Telonics, USA (Table S1). Individuals were released in open waters, close to the island of Madeira (Fig. 1). The tracking period lasted from a minimum of 56 days to a maximum of 342 days, with an average of 226 ± 105 days (mean ± sd, Table 1-S1).

### Strandings and sightings data

The datasets of stranding records of loggerhead sea turtles used for validation includes records in Irish, UK and French waters. Irish and UK records [61] are freely available on Ocean Biodiversity Information System (OBIS, https://obis.org/). Strandings data on the French Atlantic coast have been provided by Aquarium La Rochelle [62]. These datasets include not only stranding events of loggerheads, but also sightings and captures of various species of sea turtles. For this study, only stranding records of loggerhead sea turtles recorded between 1998 and 2022 were used.

### Data filtering and processing

Raw locations derived from Argos-linked PTTs were used for the interpolation and filtering process without the need of previous steps, while data obtained from geolocators required additional processing. Raw data from the geolocators was visually checked using the IGOR Pro software (WaveMetrics Inc., RRID: SCR_000325). A total of 19 geolocators were excluded from further analysis, because they recorded no (11 tags) or few light level data (8 tags). Next, the GPE3 model (Wildlife Computer Inc.) was used to obtain locations based on light levels, SST and Argos monthly locations. This model fits a state space model (SSM) allowing the user to define the time interval between subsequent locations (24 h) and the maximum speed of the animal (2.5 m/s based on published literature, e.g. [28]). Once we obtained the SSM fitted tracks, only the corrected location estimates were used for further analysis.

The subsequent processing of data and habitat modelling were conducted using R Statistical Software (v4.2.3; R Core Team 2023). All the tracks, both from Argos linked PTTs and geolocators, were then interpolated using the aniMotum R package (version 1.1, [63]). The package was used to fit a SSM for each individual, with estimated locations every 24 h and allowing a maximum speed of 2.5 m/s. The SSM uses the Argos location quality as an estimate of the error of locations obtained from PTTs, while it needs to be supplied with longitudinal and latitudinal errors for geolocators. The values used were 1º and 1.8º, respectively, and were derived from previous studies [64, 65]. The R package pathroutr (version 0.2.1, [66]; now part of aniMotum) was then used to correct the tracks for land avoidance. To avoid artifacts generated by the SSM in the interpolation process, time gaps greater than 10 days between two subsequent raw locations were identified. The interpolated tracks were then divided into segments to avoid those time gaps. Only segments of a minimum of 5 subsequent locations where retained.

These segments were then used to generate correlated random walks (CRWs), to be used as pseudo-absence data in the model. 100 CRWs were generated from each segment following [46, 51]. Simulated positions on land were excluded from the pseudo-absence dataset.

Since individuals tagged in the Canary Islands were released from shore [30], the first 7 days of tracking were excluded from both tracking data and CRWs. Cross-basin migrations (defined as westward and persistent movements) were also identified and excluded. Tracks were visually checked for directional and persistent movement towards the West Atlantic coasts. Only 12 individuals (IDs 10340, 10341, 10343, 22209, 23662, 24186, 24189, 78457, 82791, 82798, 87501, 94949) showed a persistent cross-basin movement. The aniMotum R package was then used to fit a movement persistence model (MPM). The coefficient estimated by the MPM model was used to determine the starting dates of the cross-basin migrations [67, 68]. These migratory segments were excluded from the tracks and none of these decisions resulted in excluding a full track.

An estimate of the active swimming speed of the turtles between subsequent locations has been calculated by subtracting the current speed (resultant of the eastward and northward sea water velocity, respectively U and V, extracted from https://doi.org/10.48670/moi-00148) to the overall speed. A maximum value of 1.67 m/s was obtained. Consequently, a value of 2 m/s was used as threshold to exclude CRWs that would have required a greater and unrealistic active swimming speed.

A set of 17 environmental variables was extracted for all the locations of the real tracks and the CRWs. These covariates were both abiotic (e.g. SST, sea level anomaly, normalised distance to the closest anticyclonic and cyclonic eddy) and biotic (e.g. chlorophyll-a concentration, net primary productivity). Selection of the covariates was based on their documented influence on sea turtle movements (e.g. [23, 37, 69–71]). All the variables were extracted at a resolution of 0.25 degree grid cell size, with the exception of the normalised positions of the closest cyclonic and anticyclonic eddies, which are based on the position of the cyclonic and anticyclonic eddies that are the closest to each location. Details about all the environmental variables used can be found in Table 2.

**Table 2:** Environmental variables used as covariates in the habitat model. Normalised positions of the closest cyclonic and anticyclonic eddies have been obtained dividing the distance to the closest cyclonic and anticyclonic eddy by the radius of the corresponding eddy. Only long-lived eddies, defined in the AVISO product as eddies that have been detected for more than 10 days, have been used

Variable	Unit	Resolution	Source	Product
Bathymetry	m	0.017°	10.7289/V5C8276M	ETOPO1
Rugosity	m	0.017°	10.7289/V5C8276M	Derived from ETOPO1
Absolute Dynamic Topography	m	0.125°	10.48670/moi-00148	SEALEVEL_GLO_PHY_L4_MY_008_047
Absolute Dynamic Topography Standard Deviation	m	0.125°	10.48670/moi-00148	Derived from SEALEVEL_GLO_PHY_L4_MY_008_047
Sea Level Anomaly	m	0.125°	10.48670/moi-00148	SEALEVEL_GLO_PHY_L4_MY_008_047
Sea Level Anomaly Standard Deviation	m	0.125°	10.48670/moi-00148	Derived from SEALEVEL_GLO_PHY_L4_MY_008_047
Mixed Layer Depth	m	0.083°	10.48670/moi-00021	GLOBAL_MULTIYEAR_PHY_001_030
Sea Surface Temperature	°C	0.05°	10.48670/moi-00168	SST_GLO_SST_L4_REP_OBSERVATIONS_010_011
Sea Surface Temperature Standard Deviation	°C	0.05°	10.48670/moi-00168	Derived from SST_GLO_SST_L4_REP_OBSERVATIONS_010_011
Mass concentration of chlorophyll-a in sea water	mg/m3	4 km	10.48670/moi-00281	OCEANCOLOUR_GLO_BGC_L4_MY_009_104
Mass content of epipelagic micronekton	g/m2	0.083°	10.48670/moi-00020	GLOBAL_MULTIYEAR_BGC_001_033
Net primary productivity of biomass	mg/m2/day	0.083°	10.48670/moi-00020	GLOBAL_MULTIYEAR_BGC_001_033
Mass content of zooplankton	g/m2	0.083°	10.48670/moi-00020	GLOBAL_MULTIYEAR_BGC_001_033
Finite-time Lyapunov exponent based on the maximum eigenvalue of the Cauchy-Green strain tensor	1/days	0.04°	10.24400/527896/a01-2022.002	SSALTO/DUACS FSLEs and Orientations of the associated eigenvectors
Eddy Kinetic Energy	m2/s2	0.125°	10.48670/moi-00148	Derived from SEALEVEL_GLO_PHY_L4_MY_008_047
Normalised position of the closest cyclonic eddy	-	Not gridded	10.24400/527896/a01-2022.005	Derived from Mesoscale Eddy Trajectories Atlas Product META3.2
Normalised position of the closest anticyclonic eddy	-	Not gridded	10.24400/527896/a01-2022.005	Derived from Mesoscale Eddy Trajectories Atlas Product META3.2

### Habitat model

A preliminary data exploration was performed to visualize distribution frequencies (boxplots and histograms, Fig. S2 – S3 – S4, Tab. S2) [72]. BRTs were then used to build and test several habitat models. All the models were built using the R packages dismo (version 1.3-9, [73]), and gbm (version 2.1.8.1, [74]), following the guidelines by [44]. BRTs are non-parametric models, defined by four parameters: number of trees, tree complexity, learning rate and bag fraction. Different combinations of these parameters create individual models that need to be evaluated to choose the best possible model. Due to the lack of independent data, a multi-pronged cross-validation based approach has been used for testing the models. Three evaluating functions were used for each model. First, the entire dataset was used both for training and testing (100%). Secondly, 75% of the dataset was used for training the model, while the remaining 25% was used for testing. Finally, we used a k-fold cross validation function (k = 10) that randomly holds 10% of the data back for testing 10 times. Several candidate models were evaluated and compared in terms of both explanatory power, using pseudo-R-squared (pseudoR2) and root mean squared error (RMSE), and predictive skill, using area under the receiver operating characteristic curve (AUC), concordance index (C-index) and ratio between predicted and observed values.

Since the ratio of presence and pseudo-absence data have an impact on the model [46, 75], the first tests were used to determine the ratio that worked best with the dataset. Five ratios (1:1, 1:2, 1:3, 1:4, 1:5) were used to build models with fixed values of learning rate (0.01) and tree complexity (5). Individual pseudo-absence locations were randomly selected from the entire CRWs dataset. The model using the 1:1 ratio had the best value of AUC, ratio between predicted and observed values, pseudo-R-squared, RMSE and C-index, therefore the subsequent tests were conducted using this ratio. Multiple combinations of learning rate values (0.0001, 0.0005, 0.001, 0.005, 0.01, 0.05, 0.1, 0.3, 0.5) and tree complexity values (3, 4, 5) were tested and compared. All models were built using a fixed value of bag fraction (0.6) and with the best number of trees determined by the “gbm.step” function from the gbm R package.

### Predictions of habitat suitability

Daily values of the environmental variables used in the model (Table 2) were extracted in a 0.25º cell grid for the period 1998–2022 for the study area (75ºW-10ºE longitude and 10ºN-60ºN latitude).

The best model obtained (see Results) was used to make daily predictions of habitat suitability for the study area. Seasonal maps were obtained by calculating the mean value of habitat suitability for each grid cell and for each season. Seasons were defined as follows: spring March-May, summer June- August, autumn September-November and winter December-February. The use of daily (contemporaneous) environmental variables has been chosen over seasonal mean values (climatological) to better capture mesoscale variability [76]. Moreover, this approach is more appropriate for the development of future dynamic management tools and the modelling of possible distribution shifts linked to climate change scenarios [76].

Persistence of habitat suitability was calculated for each grid cell as the proportion of days in which the predicted habitat suitability was higher than 0.5. The threshold value used (0.5) is the upper quartile (0.75) of the distribution of habitat suitability values obtained for the study area during the period 1998–2022, after excluding the lowest values of habitat suitability (< 0.1). Persistence values and seasonal persistence values were calculated for each year of the predicted period and for the entire period.

## Results

### Tracking data

After the filtering process, 162 segments of tracking data were obtained from 105 individuals, 76 tagged with Argos linked PTTs and 29 with geolocators (Fig. 1). Most of the locations (~ 72%) were collected after the year 2000 (Fig. S2A), while the monthly distribution of the locations is more uniform (Fig. S2B). The duration of the tracks’ segments spanned from 5 days to 753 days. Most of the individuals released in the Azores moved around the archipelago, in a circular area of approximately 500 km of radius (Fig. 1). One turtle (ID 2854) went further than the others, reaching almost 50° N and then moving eastward before going southward (Fig. 1). Turtles released in the Canary Islands mainly moved along the North-West African coast, reaching the Portuguese and Spanish coasts to the North and Mauritania to the South (Fig. 1). One individual (ID 94955) moved to the North-West of the basin, reaching the waters West of the Azores with convoluted movements (Fig. 1). This last trend was more common among turtles tagged in Madeira, of which 5 out of 10 moved north-westward, while the others moved along the African coast (Fig. 1). Turtles tagged in the Central North Atlantic mainly moved eastward, while 8 individuals moved southward and then towards the North American coast (Fig. 1).

### Habitat model

**Fig. 2:**
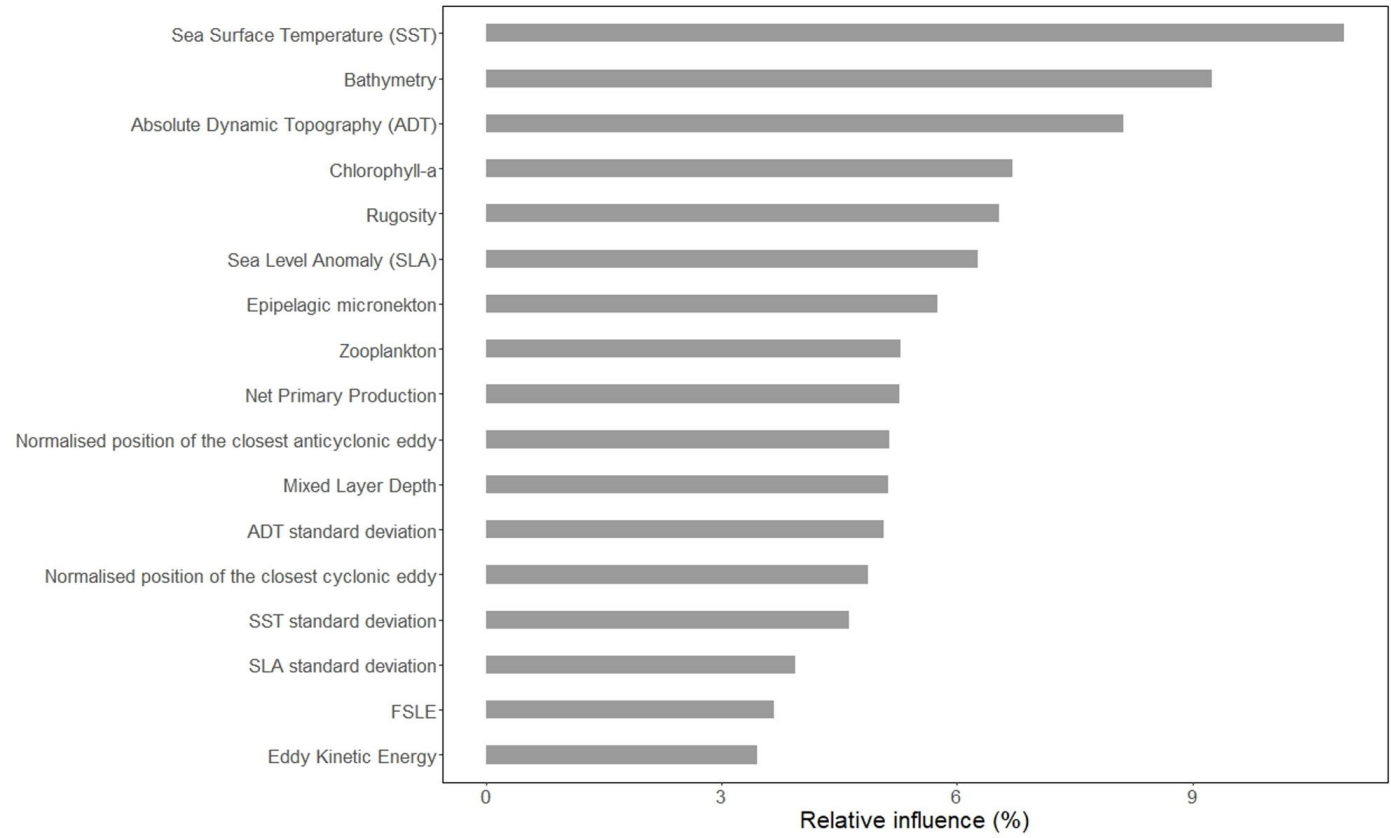
Influence of the environmental variables. Relative influence of each environmental variable on the output of the best model obtained

The best model obtained, in terms of predictive power, used a dataset with a presence/pseudo-absence ratio of 1:1. Presence and pseudo-absence locations used in the final model are shown in Fig. S5. The model parameters selected were 0.1 for learning rate and 5 for tree complexity, with the best number of trees set at 5700. The best model showed good predictive power (Table 3), and all the environmental variables used showed a relative influence different from 0 (Fig. 2). The most relevant variable was SST (10.94%, Fig. 2) and the 7 most influential variables (SST, bathymetry, absolute dynamic topography, chlorophyll-a concentration, rugosity, sea level anomaly and epipelagic micronekton concentration) summed to more than 50% of relative influence (Fig. 2).

**Table 3:** Model evaluation parameters. Parameters used were: area under the receiver operating characteristic curve (AUC), ratio between predicted and observed values (PRED/OBS), pseudo-R-squared (pseudoR2), root mean squared error (RMSE) and concordance index (C-index). Values in this table are relative to the best model obtained. These parameters have been calculated using a 75/25 and a 100/100 ratios of the dataset for, respectively, training and testing. Last row of the table shows the mean of the two values obtained

Evaluation function	AUC	PRED/OBS	pseudoR2	RMSE	C-index
75/25	0.984	0.997	0.541	0.339	0.939
100/100	0.992	1	0.599	0.269	0.992
-	0.988	0.999	0.57	0.304	0.966

### Predictions of habitat suitability

**Fig. 3:**
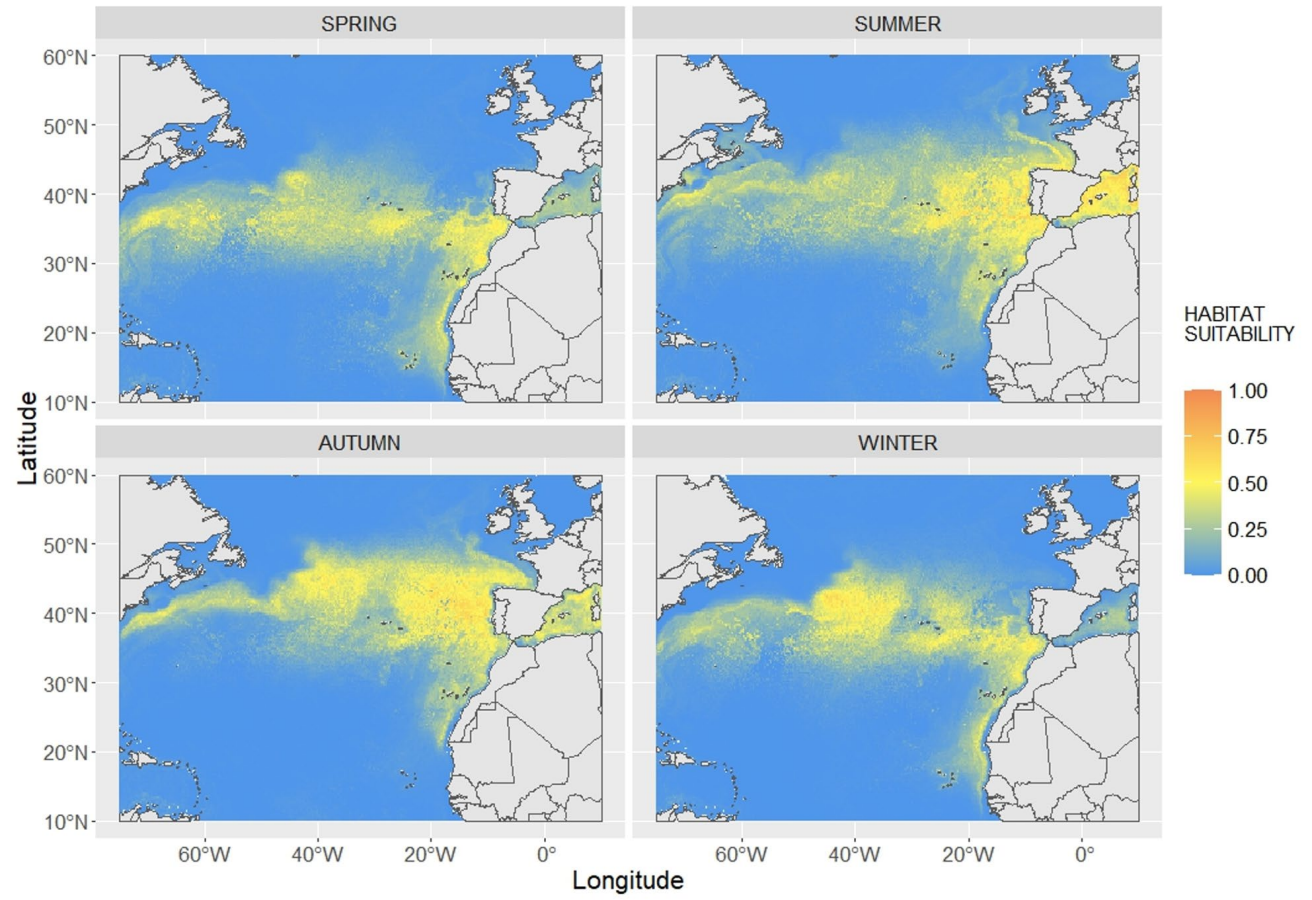
Seasonal predictions of habitat suitability for the study area. Values shown represent the mean of the predictions made for each 0.25°x0.25° cell for the period 1998–2022. Seasons are defined as spring March-May, summer June- August, autumn September-November and winter December-February

**Fig. 4:**
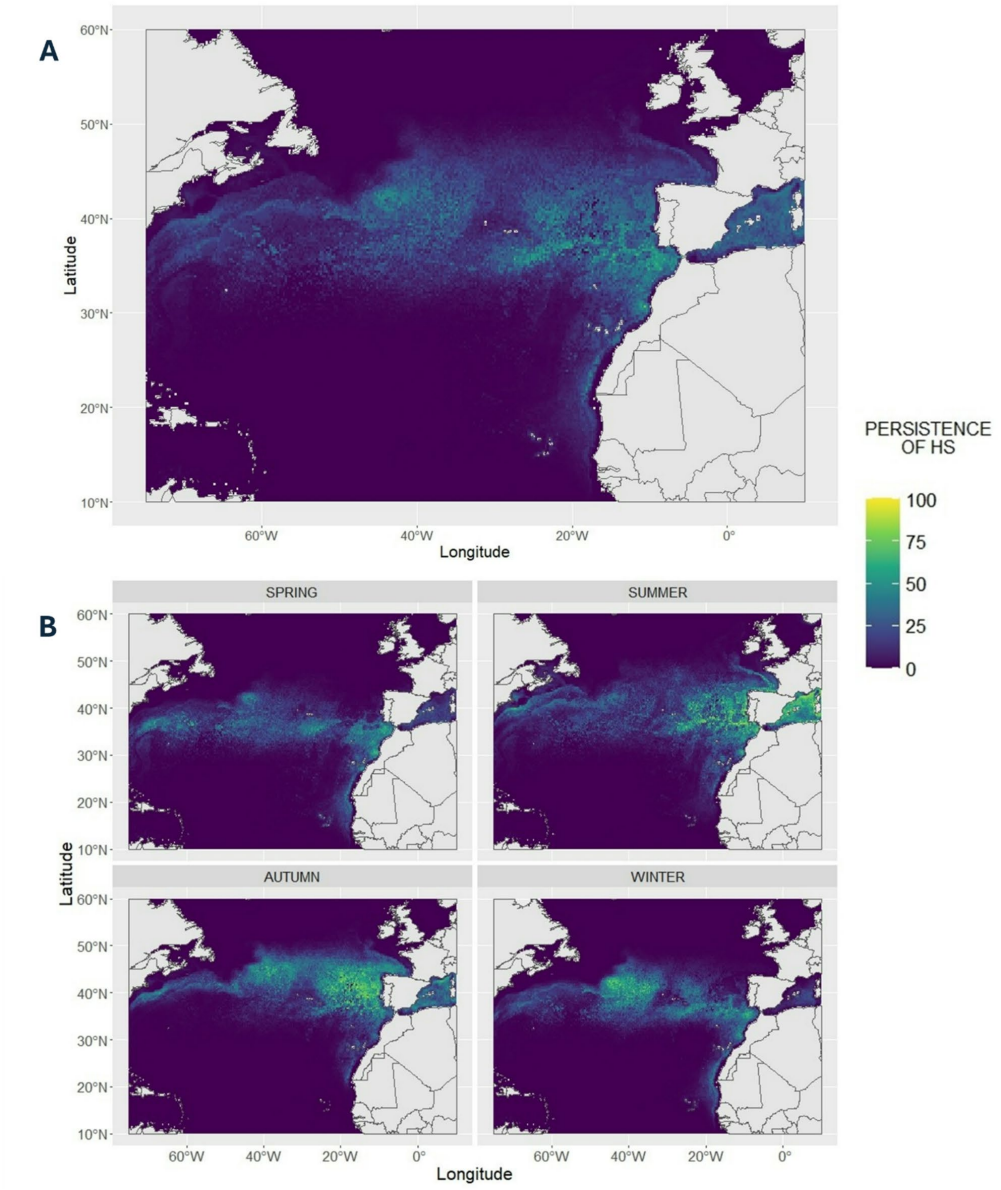
Persistence of habitat suitability in the study area. The value of persistence of habitat suitability represents the percentage of days in which habitat suitability was higher than 0.5. **A**: persistence calculated over the entire predicted period (1998–2022). **B**: persistence for the same period but divided by season. Seasons are defined as spring March-May, summer June- August, autumn September-November and winter December-February

Daily predictions of habitat suitability showed a persistent latitudinal area of high habitat quality that goes from the West to the East of the basin (Figs. 3 and 4A). The southern limit of this band does not shift seasonally and is persistent around 30º N of latitude (Figs. 3 and 4B). South of this horizontal band the values of predicted habitat suitability are close to 0. This area of poor habitat suitability extends from the western limit of the study area through the waters around Cape Verde and does not vary across seasons (Figs. 3 and 4B).

**Fig. 5:**
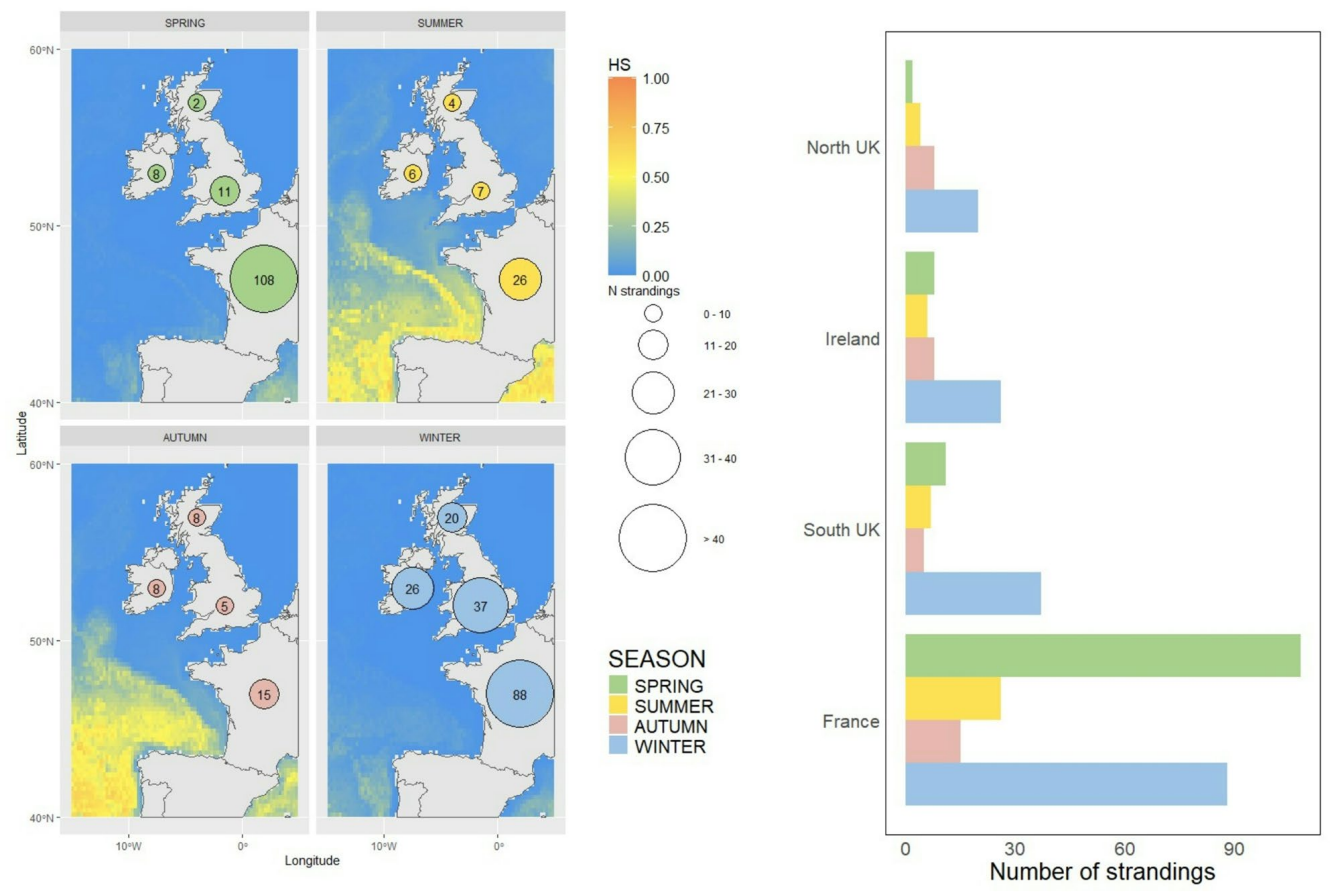
Stranding events and habitat suitability. Stranding records of both dead and alive loggerheads in the French Atlantic coast, Ireland and UK during the predicted period (1998–2022), overlapped with seasonal habitat suitability predictions for the area (15ºW-5ºE longitude and 40ºN-60ºN latitude). Seasonal counts of strandings events for the period 1998–2022 in each area are shown in the bar plot

Conversely, the northern limit of suitable habitat is highly seasonal (Figs. 3 and 4B). During warmer seasons (summer and autumn) it moves northward (about 600 km) including the coastal waters of Ireland, UK, France and part of the North Sea. Winter and spring, in contrast, show a compressed distribution of good habitat suitability, which is limited at around 45º N. This seasonal pattern is conspicuous in the Bay of Biscay, which is predicted to have higher habitat suitability for juveniles in summer-autumn than during winter-spring (Fig. 5).

Waters surrounding the Canary Islands are predicted to be good for juveniles year round, like most of the area along the West African Coast. Seasonality is evident again in the Cape Verde area, which is predicted to have better habitat during winter-spring than during summer-autumn. The western Mediterranean Sea included in the study area (waters West of Sardinia and Corsica) is predicted to be optimal during summer and autumn, while it is less suitable for juveniles during spring and winter (Fig. 3).

### Strandings and sightings

A total of 379 stranding events of both dead and alive loggerheads has been recorded in the Atlantic French coast and in UK and Irish waters during the predicted period (1998–2022), with the majority of the strandings (62.5%) happening in France (Fig. 5). Most of the strandings happened during spring and winter, respectively 34.1% and 45.1% of the recorded events, when habitat suitability is predicted to be lower than during summer and autumn, when only 11.3% and 9.5% of the stranding events occurred. Moving among the four areas considered from South to North (France, South UK, Ireland and North UK), the percentage of stranding events on the total decreases progressively (France 62.5%, South UK 15.8%, Ireland 12.7%, North UK 9.0%; Fig. 5) and only in the French coast the majority of strandings was recorded in spring (45.6%), while in the northern areas more than 50% of the strandings happened in winter (Fig. 5).

## Discussion

This study represents the first effort to merge multiple datasets of oceanic juvenile loggerhead sea turtle movements in the North Atlantic and provides the first habitat model for this life stage in the entire basin. Thanks to the collaboration across several research teams, the dataset of tracking data collected covered the entire North Atlantic to provide fundamental insights of the habitat of juvenile loggerheads. Predictions obtained using the developed model highlighted areas of high and persistent values of habitat suitability and showed the seasonal variations of habitat suitability in the basin.

The well-known influence of the Gulf Stream on megafauna movements (e.g. [70, 77, 78]). as well as the importance of the Azores current is represented in the model. The Azores current is a branch of the Gulf Stream which moves from the Gulf Stream Bifurcation (approximately 40°N, 45° W) until the eastern part of the North Atlantic [79, 80]. The Azores current is located between 33ºN and 36ºN of latitude [80], which overlaps with the southern limit of the good habitat area predicted by our model (Figs. 3 and 4). This important influence of the major currents on the movements of juvenile (and hatchling) loggerheads has been shown also in the Pacific Ocean, where the Kuroshio and the North Pacific Current are fundamental during the eastward migration of juveniles [34–36, 38].

The predictions and persistence maps highlight areas that show high habitat suitability values all year round: the horizontal band between 30 and 45° N (Figs. 3 and 4). This area includes regions that are well-known developmental habitat for oceanic juvenile loggerheads: the Azores [6, 57, 81, 82], the waters in front of the Iberian coast [10, 12, 83, 84], Madeira [29, 81, 85], the Canary Islands and the coastal waters of West Africa [30]. Even if the Azores are included in the favourable horizontal area, predictions for the waters surrounding the archipelago are not as good as expected from previous studies regarding this area (Figs. 3 and 4). A possible explanation for this mismatch in shallower oceanic areas (< 2000 m) is the high influence of bathymetry on predictions (9.24%, Fig. 2), which is evident West of the Azores and in the Bay of Biscay, where the shape of the Mid Atlantic Ridge and the canyons in the Bay of Biscay are clearly visible (Fig. 3). Moreover, most of the presence locations (82.32%, Fig. S3) were in waters deeper than 2000 m. Low coverage of shallower waters and the great influence of bathymetry may have led to the underestimation of habitat suitability in the Azores, where sightings are documented to occur close to shore [82].

The favourable area predicted by the model is wider in terms of latitudinal span than the favourable area predicted for juveniles in the North Pacific ([23, 69]). Turtles latitudinal movements in the North Pacific have been linked to the transition zone chlorophyll front, since turtles movements follow its seasonal latitudinal shift [35, 37]. Our predictions show a stable southern limit of the suitable habitat around 30º N, while the northern limit of the distribution is subject to seasonal changes, from about 45º N in spring to more than 50º N in summer (Fig. 3). This seasonal pattern is stronger in the eastern side of the basin, were the seasonal difference in terms of latitudinal span is more evident (Fig. 3). The Bay of Biscay and the UK and Irish waters are a good example of this seasonal trend (Fig. 5). The strong seasonality of predicted habitat suitability is consistent with observed stranding patterns in the area during the predicted period ([13, 14]; Fig. 5). In fact, the number of stranded loggerheads in the Atlantic French coast, UK and Ireland are higher during winter and spring, seasons in which the model presented in this study predicts low habitat suitability values in the area (Fig. 5). Moreover, it appears that most of the strandings happen mostly close, both temporally and spatially, to the Northern limit of the favourable habitat (Figs. 3, 4 and 5). The fact that strandings are more frequent when the persistence of good habitat is lower (Figs. 4 and 5) may indicate that a longer season of good habitat may be important in determining the survival of individuals. As stated in the Results, France showed a higher percentage of strandings during spring ([62]; Fig. 5), while in the other areas considered most of the strandings occurred in winter ([61]; Fig. 5). Waters in the Bay of Biscay may allow the survival of part of the individuals through winter, with a consequent higher number of strandings happening after winter and during spring, when individuals have faced a longer period of time in less suitable habitat. However, the poor conditions of alive stranded individuals shed doubts on the actual chance of survival in this colder waters [86]. In Irish and UK waters, at northern latitudes, survival through winter may be more difficult, probably because of lower SST, resulting in a higher percentage of strandings occurring during this season.

While strandings data can be useful to assess the realism of the model output near the fringes of the animals’ distribution, they may not be as explanatory for areas that are predicted to be good all year around. Bycatch rates may be more directly applicable for this validation. Moreover, bycatch is the main threat encountered by loggerheads in the North Atlantic [22, 87], thus our model can provide important insights on loggerheads distribution for management and conservation purposes. Bycatch of loggerheads has been reported from Canadian and US fishing vessels near the Gulf Stream, areas that our predictions show to be good for juveniles [21, 24, 27]. In these areas, higher bycatch rates have been reported for the period between July and December [27]. This same pattern in bycatch rates has been observed for the Portuguese pelagic longline fishery operating mainly between the Azores and mainland Portugal [25]. This study also highlighted how bycatch was clustered between Portugal and the Azores during autumn and West of the Canary Islands during summer [25]. Our model predicts those areas to have high values of habitat suitability during those seasons (Fig. 3). However, it’s interesting to note that, both West of the Azores and between the Azores and Portugal, the predictions do not match the seasonality observed in bycatch rates. In fact, in the Northwest Atlantic the habitat suitability is persistently high throughout the year (Fig. 4), while between Portugal and the Azores, the habitat suitability is higher during both summer and autumn (Fig. 3), and it shows a certain degree of persistence all year around (Fig. 4). This suggests that habitat suitability is not the sole factor determining the interaction between juvenile loggerheads and fisheries. Several studies have shown that operational factors have an impact on bycatch (e.g. [88, 89]), but it is possible that additional environmental parameters (e.g. food availability) should be considered while assessing the risk of bycatch. Our predictions may constitute a robust baseline to further investigate where and when juvenile loggerhead sea turtles are most likely to interact with fishery and help the development of bycatch mitigation policies in potential high use areas of loggerheads.

Extrapolated habitat predictions in Cape Verde and in the Mediterranean Sea should be interpreted with care. It is known that individuals of these populations mix with individuals from North East of the basin [90–92], but their dispersal movements and developmental areas may be different. Cape Verde is a well known nesting area for loggerhead sea turtles, totalling more than 10,000 nests per year in the archipelago [4]. Genetic studies have shown that juveniles from the Cape Verde rookeries spread towards the Canary Islands, the Azores but also in the western Mediterranean Sea [17, 19, 92], while an estimate of 43% of individuals spend their lost years in unknown areas [92]. Our model predicts the area around Cape Verde to have low values of habitat suitability. This poor habitat suitability can be a possible explanation for the high percentage of juveniles from Cape Verde migrating to more favourable areas. The Mediterranean Sea also hosts a different population of loggerheads, but mixing with the North Atlantic has been documented by several genetic studies (e.g. [17, 84]). Our model predicts a high level of persistence of good habitat suitability in the western Mediterranean (Fig. 4), showing that the environmental characteristics in this area are suitable for juveniles from around the North Atlantic. This model output agrees with the distribution of Atlantic juveniles in the western Mediterranean [93, 94].

Making habitat suitability predictions was the main goal of this study, but it is interesting to briefly explore the relationship between environmental variables and habitat quality. It is not a surprise that SST was the most influential variable (Fig. 2), since many studies have highlighted its importance for sea turtles [30, 34, 69, 71, 95]. As mentioned, only 7 out of 17 variables contribute for more than 50% to the final output (Fig. 2). It is important to acknowledge that this apparent “dilution” of variable importance across multiple predictors may be partially attributed to collinearity among covariates and the specific mechanism by which BRTs partition variance between correlated features [44, 45]. Nevertheless, it is interesting to notice that the 7 most influent variables are related to different aspects of the marine environment. Seabed characteristics (bathymetry and rugosity), SST, oceanic circulation (absolute dynamic topography and sea level anomaly) and proxies for food availability (represented by chlorophyll-a concentration and epipelagic micronekton concentration) are all important to determine the suitability of habitat for juvenile loggerhead sea turtles. Furthermore, our model shows that mesoscale eddies have an impact on loggerheads movements; in fact, the distance from both cyclonic and anticyclonic eddies has an influence greater than 0 on model output (respectively 4.86% and 5.14%, Fig. 2). Several studies have shown the impact of mesoscale eddies on loggerheads movements (e.g. [28, 36, 96]). This relationship can be particularly important in highly dynamic areas, such as the central North Atlantic. For example, a recent study [28] showed how loggerhead sea turtles tagged and released in the Azores (included in the dataset used in this study) spent more time inside older anticyclonic eddies than inside cyclonic eddies or outside any type of eddies. Eddies can have varied impact based on location and their age [97, 98], thus further studies are needed to understand why loggerheads in the North Atlantic spend more time inside eddies than outside, yet preferences for eddies may vary spatially similarly to what has been observed in the Pacific Ocean [36]. Individual-based analyses that explore attraction and dispersal around eddies can be an important next research avenue.

While BRTs have proved to be an appropriate tool for making predictions of habitat suitability for the entire basin, they are not the best option when it comes to understanding the relationship between environmental variables and habitat suitability [44]. Further investigations with parametric modelling frameworks could yield more insights on how they interact with each other and affect habitat suitability. However, BRTs have consistently been shown to have high predictive skill relative to other modelling approaches which was a priority of this study [44].

Overall, BRTs proved to be a good framework to model the habitat preferences of juvenile loggerheads in the North Atlantic as this technique allowed us to use the entire tracking dataset (almost 20000 entries only of presence data; Fig. S5) and to use all potential environmental covariates that might impact turtles movements, while obtaining a model with robust predictive power (Table 3). However, it is important to remember that habitat models predict potential habitat that may not correspond to realized habitat. The predictions calculated in this study (Fig. 3) are fundamental to have a broader idea of where the habitat is more suitable for loggerheads, but other factors like population abundance, currents and life history (researchers have suggested that loggerheads may return to favourable habitat that they have already visited, like the Azores; e.g. [6]). should be considered before translating these results into estimate of species density and subsequent management actions [47, 51, 99]. Nevertheless, our model represents an important step for understanding the preferences of juvenile loggerheads in the North Atlantic.

## Conclusions

The habitat model for juvenile loggerhead sea turtles in the North Atlantic presented in this study has good predictive power (Table 3) and has high biological realism when compared to reports of stranded animals (Fig. 5) and bycatch rates. Environmental variable preferences are similar to previous studies indicating the importance of SST [58, 100] and oceanographic features like mesoscale eddies [28, 97, 98]. The areas that appear to be persistent all year around (Fig. 4) are well known habitats of juveniles and, all together give us a first cumulative map of their potential distribution and help us identify important areas for loggerheads [101]. This is crucial information for advancing management, conservation and protection of the species critical life history stages in the North Atlantic. As mentioned previously, most existing studies have had a regional focus while the threats to these animals are distributed in the entire basin. This joint model allows a more holistic understanding of their habitat and distribution.

This is only the first step needed to develop tools that will help inform researchers and stakeholders on the presence of juvenile loggerheads in the North Atlantic and, hopefully, move towards a dynamic and flexible management of the threats that this species faces, following the precedent set in the Pacific Ocean [23]. Further studies are needed to understand at a finer scale the relation between environmental characteristics, operational factors and human uses of the ocean. Nonetheless, this habitat model represents the scaffolding to advance our understanding of these relationships at the basin scale.

## Supplementary Information

Below is the link to the electronic supplementary material.

Supplementary Material 1: Additional file 1: Figure S1. Distribution of CCL of the individuals. Figure S2. Temporal distribution of the presence data. Figure S3. Distribution of the environmental variables for presence data. Figure S4. Distribution of the environmental variables for presence and pseudo-absence data. Figure S5. Presence and pseudo-absence data. Table S1. Details about individuals’ size, tagging location and time, type of tag used and capture condition. Table S2. Values of the environmental variables for presence data

## Data Availability

Loggerhead sea turtles movement datasets used in this study have been previously published and inquiries about them should be addressed to the corresponding owners. Information about the codes used should be asked to the corresponding author. After publication we plan to make predictions of habitat suitability available.

## Abbreviations

AUC: Area under the receiver operating characteristic curve
BRT: Boosted regression tree
CCL: Curved carapace length
C-index: Concordance index
CRW: Correlated random walk
GAMM: Generalised addictive mixed model
GLMM: Generalised linear mixed model
MPM: Movement persistence model
OBIS: Ocean Biodiversity Information System
pseudoR2: Pseudo-R-squared
PTT: Platform transmitter terminal
RMSE: Root mean squared error
SSM: State space model
SST: Sea surface temperature
