# Supplementary material for: Juveniles and where to find them: a basin-scale habitat model for the lost years of loggerhead sea turtles in the North Atlantic
Source: Mov Ecol. 2026 Mar 14;14:22. doi: 10.1186/s40462-026-00640-2 (PMC13045095; doi:10.1186/s40462-026-00640-2)
Supplement: Supplementary file 1 — Supplementary Material 1: Additional file 1: Figure S1. Distribution of CCL of the individuals. Figure S2. Temporal distribution of the presence data. Figure S3. Distribution of the environmental variables for presence data. Figure S4. Distribution of the environmental variables for presence and pseudo-absence data. Figure S5. Presence and pseudo-absence data. Table S1. Details about individuals’ size, tagging location and time, type of tag used and capture condition. Table S2. Values of the environmental variables for presence data [file 40462_2026_640_MOESM1_ESM.pdf]

Supplementary Materials for

Juveniles and where to find them: a basin-scale habitat model for the lost years of loggerhead sea turtles in the North Atlantic

M. Bartolini[1,2], E. L. Hazen[3,4], H. Parra[1,2], K. A. Bjorndal[5], Alan B. Bolten[5], F. Dell'Amico[6], T. Dellinger[7,8,9], R. Dietz[10], Marco A. R. Santos[11], C. Sasso[12], N. Varo-Cruz[13], J. A. Bermejo[14], D. Cejudo[13], L. F. López-Jurado[15], F. Vandeperre[1,2]

[1]Institute of Marine Sciences - Okeanos, Universidade dos Açores, Horta, Portugal

[2]Institute of Marine Research – IMAR, Horta, Portugal

[3]Ecosystem Science Division, Southwest Fisheries Science Center, National Marine Fisheries Service, National Oceanic and Atmospheric Administration (NOAA), Monterey, California, USA

[4]Institute of Marine Sciences, University of California Santa Cruz, Monterey, California, USA

[5]Archie Carr Center for Sea Turtle Research (ACCSTR), University of Florida, Florida, USA

[6]Centre d'Etudes et de Soins pour les Tortues Marines, Aquarium La Rochelle, La Rochelle, France

[7]Centro de Investigação emBiodiversidade e Recursos Genéticos (CIBIO), InBIO Laboratório Associado, Universidade do Porto, Vairão, Portugal

[8]Estação de BiologiaMarinha do Funchal, Universidade da Madeira, Funchal, Portugal

[9]BIOPOLIS Program in Genomics, Biodiversity and Land Planning, CIBIO, Vairão, Portugal

[10]Department of Ecoscience, Arctic Research Centre, Aarhus University, Roskilde, Denmark

[11]Direção Regional de Políticas Marítimas (DRPM), Horta, Portugal

[12]National Oceanic and Atmospheric Administration (NOAA), National Marine Fisheries Service, Southeast Fisheries Science Center, Miami, Florida, USA

[13]Cetaceans and Marine Research Institute of the Canary Islands (CEAMAR), Canary Islands, Spain

[14]Observatorio Ambiental Granadilla (OAG), Canary Islands, Spain

[15]Departamento de Biologia, Universidad de Las Palmas de Gran Canaria, Canary Islands, Spain

Corresponding author: Martina Bartolini, martina.bartolini@uac.pt

Figure S1. Distribution of CCL of the individuals. CCL measurements [cm] of the tagged animals in the four tagging areas.

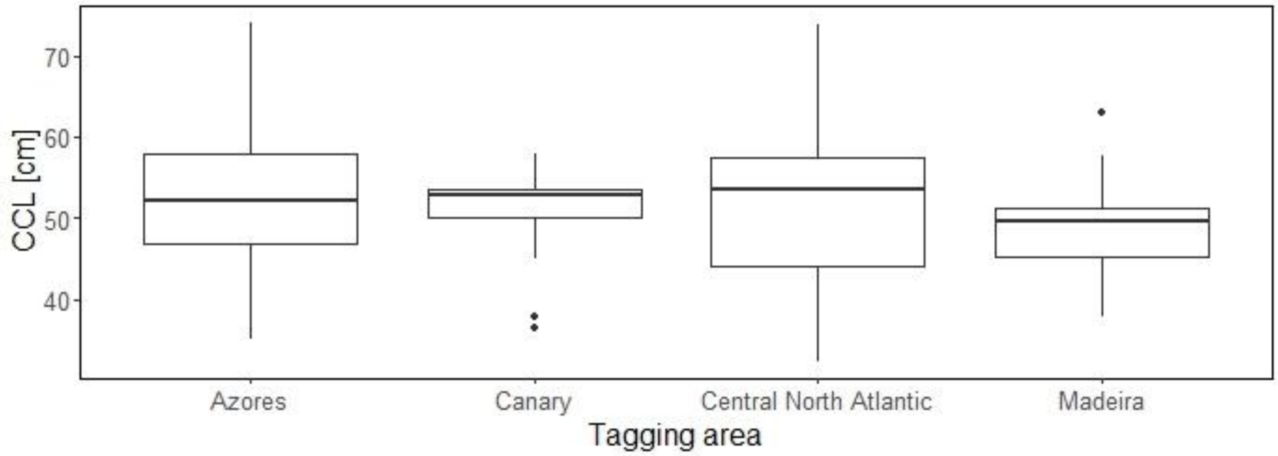

Figure S2. Temporal distribution of the presence data. Number of presence locations by year (**A**) and by month (**B**).

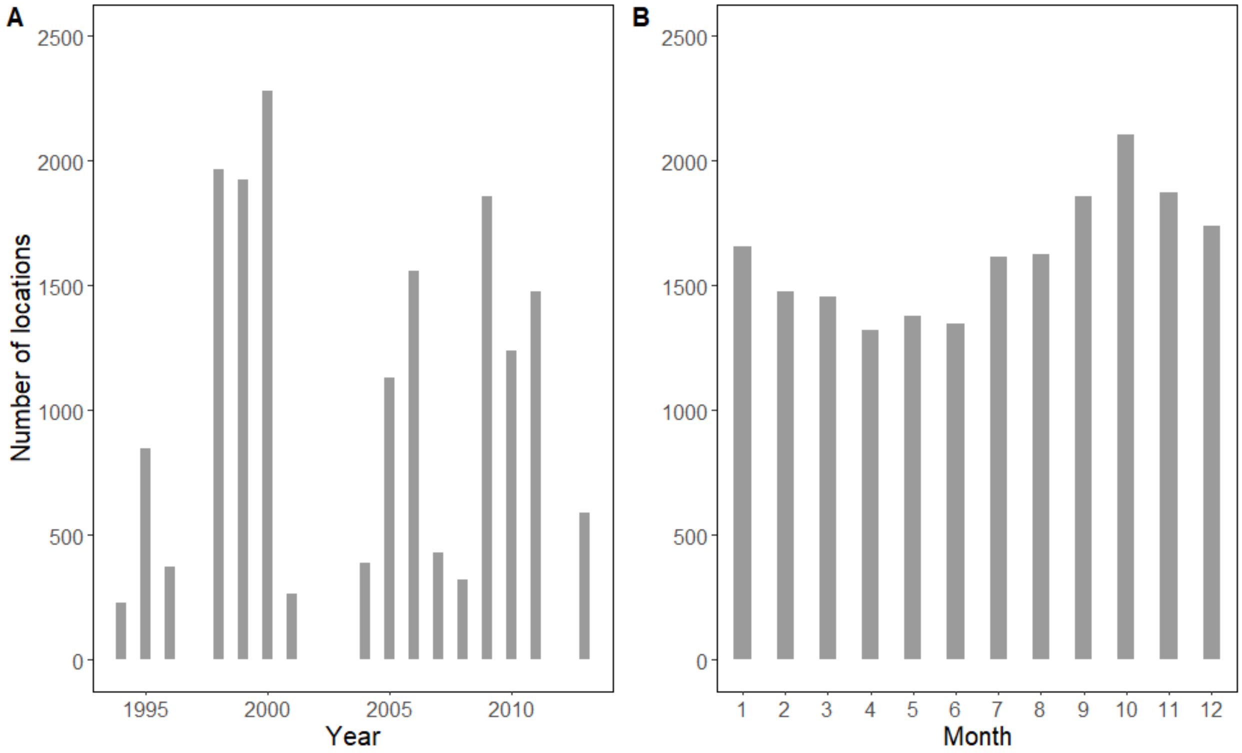

Figure S3. Distribution of the environmental variables for presence data. Histograms representing the distribution of the environmental variables for presence data. The y-axis represents the number of locations.

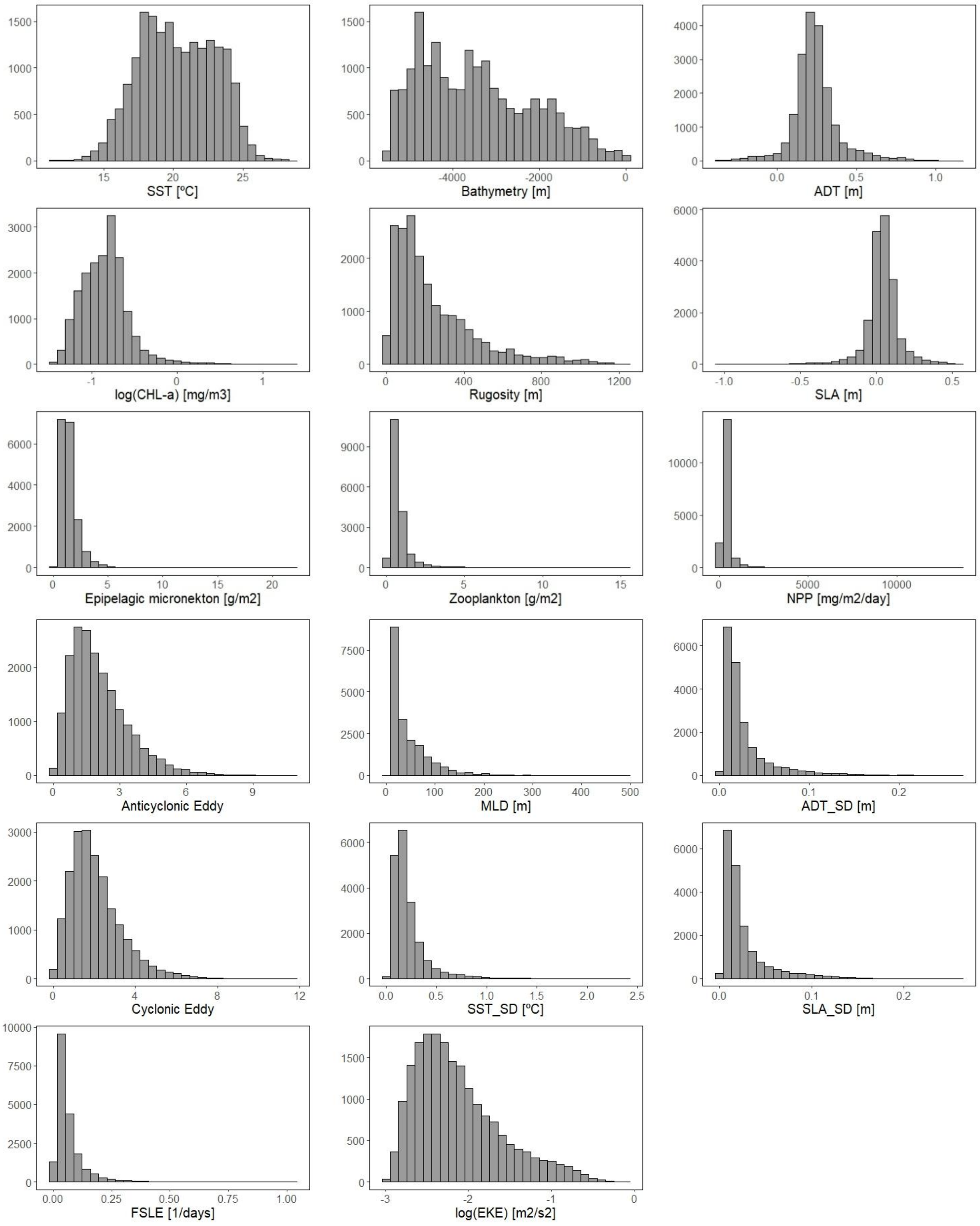

Figure S4. Distribution of the environmental variables for presence and pseudo-absence data. Boxplots representing the distribution of the environmental variables for presence (1) and pseudo-absence (0) data.

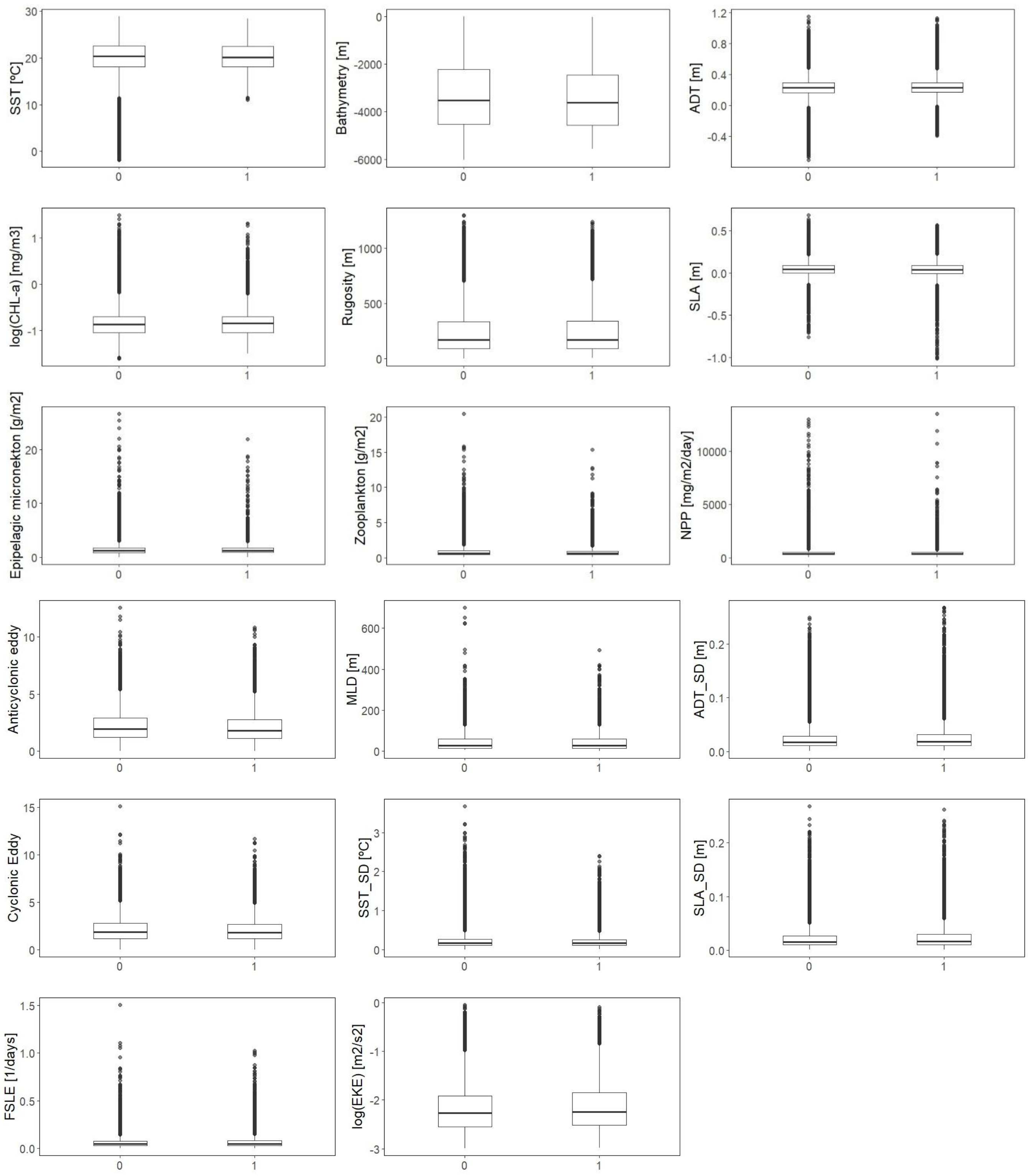

Figure S5. Presence and pseudo-absence data. Dataset of presence (blue) and pseudo-absence (black) locations used to fit the final habitat model, with a 1:1 ratio of presence/pseudo-absence locations.

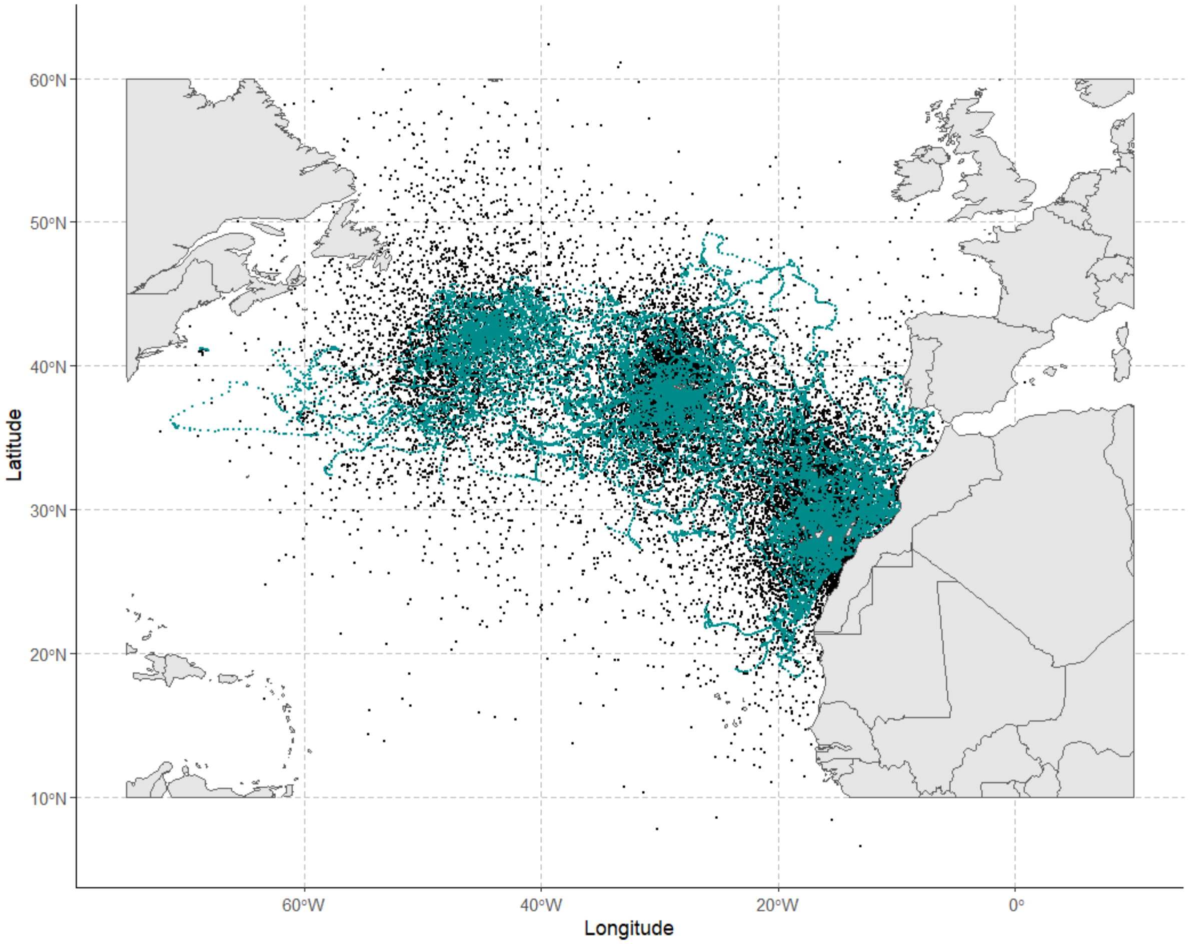

Table S1. Details about individuals' size, tagging location and time, type of tag used and capture condition.

TURTLE ID	CCL [cm]	TAG TYPE	PRODUCER	DUTY CYCLE	TAG DATE	FINAL DATE	TRACKING DAYS	CAPTURE CONDITION	AREA
**2854**	54.8	SPOT4	Wildlife Computers – USA	1 day on/1 day off	24/09/2004	30/08/2006	705	Wild	Azores
**2919**	59.5	SPOT4	Wildlife Computers – USA	1 day on/1 day off	24/09/2004	17/06/2005	266	Wild	Azores
**3758**	35.0	SPOT4	Wildlife Computers - USA	Every day on	24/09/2004	26/02/2005	155	Wild	Azores
**4188**	60.4	SPOT4	Wildlife Computers – USA	1 day on/1 day off	27/09/2004	17/06/2005	263	Wild	Azores
**6173**	63.6	SPOT4	Wildlife Computers – USA	1 day on/3 days off	29/06/2005	11/10/2005	104	Wild	Azores
**6174**	58.0	SPOT4	Wildlife Computers – USA	1 day on/3 days off	29/06/2005	21/09/2005	84	Wild	Azores
**10340**	74.0	SPOT4	Wildlife Computers – USA	1 day on/3 days off	1/07/2005	18/10/2005	109	Wild	Azores
**10341**	69.0	SPOT4	Wildlife Computers – USA	1 day on/3 days off	13/07/2005	2/01/2006	173	Wild	Azores
**10342**	58.0	SPOT4	Wildlife Computers – USA	1 day on/3 days off	2/07/2005	19/05/2006	321	Wild	Azores
**10343**	72.0	SPOT4	Wildlife Computers – USA	1 day on/3 days off	15/07/2005	18/04/2006	277	Wild	Azores

TURTLE ID	CCL [cm]	TAG TYPE	PRODUCER	DUTY CYCLE	TAG DATE	FINAL DATE	TRACKING DAYS	CAPTURE CONDITION	AREA
**20011**	46.8	ST-16	Telonics – USA	1 day on/1 day off	8/06/2000	3/10/2000	117	Wild	Azores
**20012**	59.4	ST-16	Telonics – USA	1 day on/1 day off	17/07/2000	4/07/2001	352	Wild	Azores
**20013**	46.1	ST-16	Telonics – USA	1 day on/1 day off	20/07/2000	13/01/2001	177	Wild	Azores
**20014**	47.1	ST-16	Telonics – USA	1 day on/1 day off	20/07/2000	6/12/2000	139	Wild	Azores
**20015**	47.0	ST-16	Telonics – USA	1 day on/1 day off	13/07/2000	25/11/2000	135	Wild	Azores
**20016**	51.8	ST-16	Telonics – USA	1 day on/1 day off	15/07/2000	5/01/2001	174	Wild	Azores
**20017**	49.2	ST-16	Telonics – USA	1 day on/1 day off	17/07/2000	6/06/2001	324	Wild	Azores
**20018**	47.3	ST-16	Telonics – USA	1 day on/1 day off	20/07/2000	28/11/2000	131	Wild	Azores
**22130**	52.3	ST-6	Telonics – USA	6 hours on/6 hours off	8/09/1994	21/07/1995	316	Wild	Azores
**22131**	45.0	ST-6	Telonics – USA	6 hours on/6 hours off	8/09/1994	13/06/1995	278	Wild	Azores

TURTLE ID	CCL [cm]	TAG TYPE	PRODUCER	DUTY CYCLE	TAG DATE	FINAL DATE	TRACKING DAYS	CAPTURE CONDITION	AREA
**22209**	53.3	ST-10	Telonics – USA	1 day on/1 day off	17/07/2000	18/04/2001	275	Wild	Azores
**22273**	52.1	ST-10	Telonics – USA	1 day on/1 day off	17/07/2000	28/07/2000	11	Wild	Azores
**22274**	50.0	ST-10	Telonics – USA	1 day on/1 day off	17/07/2000	25/08/2000	39	Wild	Azores
**22275**	55.0	ST-10	Telonics – USA	1 day on/1 day off	17/07/2000	21/02/2001	219	Wild	Azores
**22530**	52.7	ST-10	Telonics – USA	1 day on/1 day off	11/11/1998	9/06/1999	210	light hooked	Azores
**22531**	41.4	ST-10	Telonics – USA	1 day on/1 day off	25/09/1998	19/02/1999	147	Wild	Azores
**23657**	67.6	ST-10	Telonics – USA	1 day on/1 day off	28/10/1998	26/03/1999	149	light hooked	Azores
**23658**	46.8	ST-10	Telonics – USA	1 day on/1 day off	7/10/1998	18/03/1999	162	Wild	Azores
**23659**	48.8	ST-10	Telonics – USA	1 day on/1 day off	5/12/1998	7/05/1999	153	light hooked	Azores
**23660**	47.1	ST-10	Telonics – USA	1 day on/1 day off	17/09/1998	19/03/1999	183	Wild	Azores

TURTLE ID	CCL [cm]	TAG TYPE	PRODUCER	DUTY CYCLE	TAG DATE	FINAL DATE	TRACKING DAYS	CAPTURE CONDITION	AREA
**23661**	40.8	ST-10	Telonics – USA	1 day on/1 day off	12/10/1998	9/03/1999	148	Wild	Azores
**23662**	45.5	ST-10	Telonics – USA	1 day on/1 day off	15/09/1998	12/03/1999	178	Wild	Azores
**24179**	44.7	ST-10	Telonics – USA	1 day on/8 days off	2/09/1999	29/02/2000	180	Wild	Azores
**24185**	51.1	ST-10	Telonics – USA	2 hours on/4 hours off	15/09/1995	29/03/1996	196	Wild	Azores
**24186**	55.2	ST-10	Telonics – USA	2 hours on/4 hours off	21/09/1995	3/03/1996	164	light hooked	Azores
**24187**	55.4	ST-10	Telonics – USA	2 hours on/4 hours off	21/09/1995	2/01/1996	103	Wild	Azores
**24188**	56.1	ST-10	Telonics – USA	2 hours on/4 hours off	21/09/1995	4/06/1996	257	light hooked	Azores
**24189**	58.9	ST-10	Telonics – USA	2 hours on/4 hours off	1/10/1995	28/10/1996	393	light hooked	Azores
**24190**	58.4	ST-10	Telonics – USA	2 hours on/4 hours off	1/10/1995	13/10/1995	12	light hooked	Azores
**25317**	40.6	ST-10	Telonics – USA	1 day on/8 days off	26/09/1999	25/11/1999	60	Wild	Azores

TURTLE ID	CCL [cm]	TAG TYPE	PRODUCER	DUTY CYCLE	TAG DATE	FINAL DATE	TRACKING DAYS	CAPTURE CONDITION	AREA
**25362**	53.8	ST-10	Telonics – USA	1 day on/8 days off	19/09/1999	6/02/2000	140	Wild	Azores
**25363**	41.6	ST-10	Telonics – USA	1 day on/8 days off	19/09/1999	8/11/1999	50	Wild	Azores
	52.7 ± 8.6						191 ± 122		Azores
**2367**	53.4	ST-10	Telonics – USA	6 hours on/6 hours off	3/11/1999	26/03/2000	144	Wild	Canary
**2393**	53.4	ST-10	Telonics – USA	6 hours on/6 hours off	3/11/1999	9/08/2000	280	Wild	Canary
**2433**	52.3	ST-10	Telonics – USA	6 hours on/6 hours off	29/10/1999	25/05/2000	209	Wild	Canary
**3328**	50.1	ST-10	Telonics – USA	6 hours on/6 hours off	26/08/1999	15/08/2000	355	Wild	Canary
**3342**	53.0	ST-10	Telonics – USA	6 hours on/6 hours off	3/04/2000	5/07/2000	93	Wild	Canary
**60528**	54.0	Sirtrack Kiwisat 101	Sirtrack – New Zealand	12 hours on/12 hours off	9/06/2006	7/07/2008	759	Wild	Canary
**60529**	53.0	Sirtrack Kiwisat 101	Sirtrack – New Zealand	12 hours on/12 hours off	14/06/2006	2/11/2006	141	Wild	Canary

TURTLE ID	CCL [cm]	TAG TYPE	PRODUCER	DUTY CYCLE	TAG DATE	FINAL DATE	TRACKING DAYS	CAPTURE CONDITION	AREA
**60533**	55.5	Sirtrack Kiwisat 202	Sirtrack – New Zealand	12 hours on/12 hours off - after 3 months 6 hours on/ 6 hours off	29/03/2006	17/07/2006	110	Wild	Canary
**60534**	51.0	Sirtrack Kiwisat 202	Sirtrack – New Zealand	12 hours on/12 hours off - after 3 months 6 hours on/ 6 hours off	29/03/2006	13/10/2006	198	Wild	Canary
**60535**	55.5	Sirtrack Kiwisat 202	Sirtrack – New Zealand	12 hours on/12 hours off - after 3 months 6 hours on/ 6 hours off	6/04/2006	20/11/2006	228	Wild	Canary
**60536**	51.5	Sirtrack Kiwisat 202	Sirtrack – New Zealand	6 hours on/6 hours off - after 3 months 12 hours on/12 hours off	29/03/2006	19/12/2006	265	Wild	Canary
**60537**	54.0	Sirtrack Kiwisat 202	Sirtrack – New Zealand	6 hours on/6 hours off - after 3 months 12 hours on/12 hours off	8/06/2006	5/03/2007	270	Wild	Canary
**78455**	49.5	Sirtrack Kiwisat 101	Sirtrack – New Zealand	12 hours on/36 hours off	4/09/2008	28/09/2008	24	Wild	Canary
**78457**	53.0	Sirtrack Kiwisat 101	Sirtrack – New Zealand	12 hours on/36 hours off	15/02/2009	11/07/2009	146	Wild	Canary
**78458**	58.0	Sirtrack Kiwisat 101	Sirtrack – New Zealand	12 hours on/36 hours off	16/02/2009	7/06/2010	476	Wild	Canary
**78461**	45.0	Sirtrack Kiwisat 202	Sirtrack – New Zealand	12 hours on/36 hours off	5/09/2008	18/04/2010	590	Wild	Canary
**78462**	51.0	Sirtrack Kiwisat 202	Sirtrack – New Zealand	12 hours on/36 hours off	14/02/2009	8/11/2009	267	Wild	Canary

TURTLE ID	CCL [cm]	TAG TYPE	PRODUCER	DUTY CYCLE	TAG DATE	FINAL DATE	TRACKING DAYS	CAPTURE CONDITION	AREA
**78464**	56.0	Sirtrack Kiwisat 202	Sirtrack – New Zealand	12 hours on/36 hours off	15/02/2009	6/04/2010	415	Wild	Canary
**94949**	53.5	Sirtrack Kiwisat 101	Sirtrack – New Zealand	12 hours on/36 hours off	8/07/2009	4/05/2010	300	Wild	Canary
**94950**	48.0	Sirtrack Kiwisat 101	Sirtrack – New Zealand	12 hours on/36 hours off	28/08/2009	2/12/2010	461	Wild	Canary
**94952**	36.5	Sirtrack Kiwisat 202	Sirtrack – New Zealand	12 hours on/36 hours off	16/06/2009	27/09/2009	103	Wild	Canary
**94954**	48.0	Sirtrack Kiwisat 202	Sirtrack – New Zealand	12 hours on/36 hours off	14/08/2009	15/09/2009	32	Wild	Canary
**94955**	52.5	Sirtrack Kiwisat 202	Sirtrack – New Zealand	12 hours on/36 hours off	14/08/2009	22/03/2012	951	Wild	Canary
**94957**	37.8	Sirtrack Kiwisat 202	Sirtrack – New Zealand	6 hours on/18 hours off	9/09/2009	8/11/2009	60	Wild	Canary
	51.1 ± 8.6						287 ± 230		Canary
**82774**	41.5	Mk10	Wildlife Computers – USA	Daily light levels + Monthly Argos transmission	16/09/2009	11/09/2010	360	Wild	Central North Atlantic
**82775**	44.1	Mk10	Wildlife Computers – USA	Daily light levels + Monthly Argos transmission	21/09/2009	21/09/2010	365	Wild	Central North Atlantic

TURTLE ID	CCL [cm]	TAG TYPE	PRODUCER	DUTY CYCLE	TAG DATE	FINAL DATE	TRACKING DAYS	CAPTURE CONDITION	AREA
82776	62.4	Mk10	Wildlife Computers – USA	Daily light levels + Monthly Argos transmission	19/10/2009	31/08/2010	316	Wild	Central North Atlantic
**82777**	49.2	Mk10	Wildlife Computers – USA	Daily light levels + Monthly Argos transmission	28/11/2009	28/11/2010	365	Wild	Central North Atlantic
82778	56.2	Mk10	Wildlife Computers – USA	Daily light levels + Monthly Argos transmission	12/08/2011	11/08/2012	365	Wild	Central North Atlantic
**82779**	56.9	Mk10	Wildlife Computers – USA	Daily light levels + Monthly Argos transmission	11/08/2011	8/10/2011	58	Wild	Central North Atlantic
**82780**	53.4	Mk10	Wildlife Computers – USA	Daily light levels + Monthly Argos transmission	15/08/2009	31/08/2010	381	Wild	Central North Atlantic
**82781**	54.5	Mk10	Wildlife Computers – USA	Daily light levels + Monthly Argos transmission	15/08/2009	15/08/2010	365	Wild	Central North Atlantic
**82782**	56.7	Mk10	Wildlife Computers – USA	Daily light levels + Monthly Argos transmission	15/08/2011	14/08/2012	365	Wild	Central North Atlantic
82783	55.4	Mk10	Wildlife Computers – USA	Daily light levels + Monthly Argos transmission	11/08/2011	31/08/2011	20	Wild	Central North Atlantic
**82784**	62.6	Mk10	Wildlife Computers – USA	Daily light levels + Monthly Argos transmission	11/08/2011	10/08/2012	365	Wild	Central North Atlantic
82785	59.0	Mk10	Wildlife Computers – USA	Daily light levels + Monthly Argos transmission	12/08/2011	11/08/2012	365	Wild	Central North Atlantic

TURTLE ID	CCL [cm]	TAG TYPE	PRODUCER	DUTY CYCLE	TAG DATE	FINAL DATE	TRACKING DAYS	CAPTURE CONDITION	AREA
**82786**	55.0	Mk10	Wildlife Computers – USA	Daily light levels + Monthly Argos transmission	11/08/2011	10/08/2012	365	Wild	Central North Atlantic
82787	50.1	Mk10	Wildlife Computers – USA	Daily light levels + Monthly Argos transmission	11/08/2011	10/08/2012	365	Wild	Central North Atlantic
**82788**	54.7	Mk10	Wildlife Computers – USA	Daily light levels + Monthly Argos transmission	13/08/2011	26/09/2012	410	Wild	Central North Atlantic
82789	68.4	Mk10	Wildlife Computers – USA	Daily light levels + Monthly Argos transmission	11/08/2011	31/10/2011	81	Wild	Central North Atlantic
**82790**	55.3	Mk10	Wildlife Computers – USA	Daily light levels + Monthly Argos transmission	11/08/2011	5/07/2012	329	Wild	Central North Atlantic
**82791**	64.9	Mk10	Wildlife Computers – USA	Daily light levels + Monthly Argos transmission	15/08/2011	14/11/2011	91	Wild	Central North Atlantic
82792	61.5	Mk10	Wildlife Computers – USA	Daily light levels + Monthly Argos transmission	11/08/2011	29/02/2012	202	Wild	Central North Atlantic
**82793**	53.5	Mk10	Wildlife Computers – USA	Daily light levels + Monthly Argos transmission	12/08/2011	11/08/2012	365	Wild	Central North Atlantic
**82794**	54.5	Mk10	Wildlife Computers – USA	Daily light levels + Monthly Argos transmission	11/08/2011	10/08/2012	365	Wild	Central North Atlantic
**82795**	65.7	Mk10	Wildlife Computers – USA	Daily light levels + Monthly Argos transmission	12/08/2011	8/08/2012	362	Wild	Central North Atlantic

TURTLE ID	CCL [cm]	TAG TYPE	PRODUCER	DUTY CYCLE	TAG DATE	FINAL DATE	TRACKING DAYS	CAPTURE CONDITION	AREA
**82796**	53.0	Mk10	Wildlife Computers – USA	Daily light levels + Monthly Argos transmission	11/08/2011	10/08/2012	365	Wild	Central North Atlantic
**82797**	67.8	Mk10	Wildlife Computers – USA	Daily light levels + Monthly Argos transmission	11/08/2011	10/08/2012	365	Wild	Central North Atlantic
**82798**	61.5	Mk10	Wildlife Computers – USA	Daily light levels + Monthly Argos transmission	11/08/2011	10/08/2012	365	Wild	Central North Atlantic
**87501**	73.4	Mk10	Wildlife Computers – USA	Daily light levels + Monthly Argos transmission	11/08/2011	18/02/2012	191	Wild	Central North Atlantic
**87502**	61.0	Mk10	Wildlife Computers – USA	Daily light levels + Monthly Argos transmission	11/08/2011	10/08/2012	365	Wild	Central North Atlantic
87503	73.9	Mk10	Wildlife Computers – USA	Daily light levels + Monthly Argos transmission	15/08/2011	29/02/2012	198	Wild	Central North Atlantic
87504	55.6	Mk10	Wildlife Computers – USA	Daily light levels + Monthly Argos transmission	12/08/2011	31/01/2013	538	Wild	Central North Atlantic
**87505**	55.6	Mk10	Wildlife Computers – USA	Daily light levels + Monthly Argos transmission	15/08/2011	14/08/2012	365	Wild	Central North Atlantic
100916	42.2	MiniPAT	Wildlife Computers – USA	Daily light levels + Monthly Argos transmission	08/08/2012	-	-	Wild	Central North Atlantic
100918	41.4	MiniPAT	Wildlife Computers – USA	Daily light levels + Monthly Argos transmission	08/08/2012	-	-	Wild	Central North Atlantic

TURTLE ID	CCL [cm]	TAG TYPE	PRODUCER	DUTY CYCLE	TAG DATE	FINAL DATE	TRACKING DAYS	CAPTURE CONDITION	AREA
100919	38.0	MiniPAT	Wildlife Computers – USA	Daily light levels + Monthly Argos transmission	11/08/2011	-	-	Wild	Central North Atlantic
**100920**	32.4	MiniPAT	Wildlife Computers – USA	Daily light levels + Monthly Argos transmission	08/08/2012	3/08/2013	360	Wild	Central North Atlantic
100922	39.7	MiniPAT	Wildlife Computers – USA	Daily light levels + Monthly Argos transmission	08/08/2012	-	-	Wild	Central North Atlantic
**100923**	35.4	MiniPAT	Wildlife Computers – USA	Daily light levels + Monthly Argos transmission	11/08/2011	5/08/2012	360	Wild	Central North Atlantic
100924	43.9	MiniPAT	Wildlife Computers – USA	Daily light levels + Monthly Argos transmission	08/08/2012	-	-	Wild	Central North Atlantic
100925	36.4	MiniPAT	Wildlife Computers – USA	Daily light levels + Monthly Argos transmission	12/08/2011	-	-	Wild	Central North Atlantic
**100927**	44.7	MiniPAT	Wildlife Computers – USA	Daily light levels + Monthly Argos transmission	15/08/2011	9/08/2012	360	Wild	Central North Atlantic
**100929**	42.9	MiniPAT	Wildlife Computers – USA	Daily light levels + Monthly Argos transmission	15/08/2011	9/08/2012	360	Wild	Central North Atlantic
100930	41.1	MiniPAT	Wildlife Computers – USA	Daily light levels + Monthly Argos transmission	11/08/2011	-	-	Wild	Central North Atlantic
**100931**	46.3	MiniPAT	Wildlife Computers – USA	Daily light levels + Monthly Argos transmission	08/08/2012	3/08/2013	360	Wild	Central North Atlantic

TURTLE ID	CCL [cm]	TAG TYPE	PRODUCER	DUTY CYCLE	TAG DATE	FINAL DATE	TRACKING DAYS	CAPTURE CONDITION	AREA
100932	47.9	MiniPAT	Wildlife Computers – USA	Daily light levels + Monthly Argos transmission	11/08/2011	5/08/2012	360	Wild	Central North Atlantic
**100933**	45.0	MiniPAT	Wildlife Computers – USA	Daily light levels + Monthly Argos transmission	11/08/2011	5/08/2012	360	Wild	Central North Atlantic
**100934**	44.9	MiniPAT	Wildlife Computers – USA	Daily light levels + Monthly Argos transmission	08/08/2012	3/08/2013	360	Wild	Central North Atlantic
**100935**	34.3	MiniPAT	Wildlife Computers – USA	Daily light levels + Monthly Argos transmission	15/08/2011	9/08/2012	360	Wild	Central North Atlantic
100936	49.0	MiniPAT	Wildlife Computers – USA	Daily light levels + Monthly Argos transmission	11/08/2011	5/08/2012	360	Wild	Central North Atlantic
100937	47.0	MiniPAT	Wildlife Computers – USA	Daily light levels + Monthly Argos transmission	12/08/2011	-	-	Wild	Central North Atlantic
	51.9 ± 10.3		Wildlife Computers – USA				270 ± 155		Central North Atlantic
**12007**	44.0	ST-10	Telonics – USA	Every day on	10/09/1998	9/06/1999	272	Wild	Madeira
**12538**	37.9	ST-10	Telonics – USA	Every day on	27/05/1998	31/10/1998	157	Wild	Madeira
**12544**	45.7	ST-10	Telonics – USA	Every day on	1/04/1998	30/12/1998	273	Wild	Madeira

TURTLE ID	CCL [cm]	TAG TYPE	PRODUCER	DUTY CYCLE	TAG DATE	FINAL DATE	TRACKING DAYS	CAPTURE CONDITION	AREA
**12545**	63.1	ST-10	Telonics – USA	Every day on	18/05/1998	19/02/1999	277	Wild	Madeira
**12546**	50.5	ST-10	Telonics – USA	Every day on	18/05/1998	3/09/1998	108	Wild	Madeira
**12547**	51.2	ST-10	Telonics – USA	Every day on	27/05/1998	4/05/1999	342	Wild	Madeira
**12570**	57.7	ST-10	Telonics – USA	Every day on	10/09/1998	18/07/1999	311	Wild	Madeira
**12571**	51.2	ST-10	Telonics – USA	Every day on	10/09/1998	13/08/1999	337	Wild	Madeira
**12573**	48.6	ST-10	Telonics – USA	Every day on	10/09/1998	10/01/1999	122	Wild	Madeira
**12574**	44.9	ST-10	Telonics - USA	Every day on	10/09/1998	5/11/1998	56	Wild	Madeira
	49.5 ± 7.2						226 ± 105		Madeira

For each area, mean value and standard deviation of CCL and tracking days has been calculated. PTTs in bold have been included in the dataset used to generate pseudo-absence data and to build the habitat model. CCL was measured directly on the animal or calculated from straight carapace length following [1].

Table S2. Values of the environmental variables for presence data.

ENVIRONMENTAL VARIABLE	MIN VALUE	MAX VALUE	MEAN ± SD
Bathymetry [m]	-5567.54	-25.83	-3441.08 ± 1315.28
Rugosity [m]	3.90	1239.00	245.27 ± 218.44
Absolute Dynamic Topography [m]	-0.39	1.12	0.24 ± 0.15
Absolute Dynamic Topography Standard Deviation [m]	0.0014	0.27	0.028 ± 0.029
Sea Level Anomaly [m]	-1.01	0.56	0.040 ± 0.11
Sea Level Anomaly Standard Deviation [m]	0.0018	0.26	0.027 ± 0.027
Mixed Layer Depth [m]	1.68	492.87	44.53 ± 42.64
Sea Surface Temperature [ºC]	11.08	28.38	20.29 ± 2.70
Sea Surface Temperature Standard Deviation [ºC]	0.019	2.41	0.23 ± 0.19
Mass concentration of chlorophyll a in sea water [mg/m3]	0.031	20.48	0.19 ± 0.40
Mass content of epipelagic micronekton [g/m2]	0.0077	21.90	1.47 ± 0.94
Net primary productivity of biomass [mg/m2/day]	37.17	13526.10	439.59 ± 393.96
Mass content of zooplankton [g/m2]	0.022	15.36	0.82 ± 0.75
Finite-time Lyapunov exponent based on the maximum eigenvalue of the Cauchy-Green strain tensor [1/days]	0.00	1.03	0.068 ± 0.071
Eddy Kinetic Energy [m2/s2]	0.0010	0.80	0.0074 ± 3.31
Normalised position of the closest cyclonic eddy	0.0073	11.68	2.08 ± 1.27
Normalised position of the closest anticyclonic eddy	0.0075	10.83	2.08 ± 1.32

Mean, standard deviation, minimum and maximum values of each environmental variable associated with presence data.

REFERENCES

1. Varo-Cruz N, Bermejo JA, Calabuig P, Cejudo D, Godley BJ, López-Jurado LF, et al. New findings about the spatial and temporal use of the Eastern Atlantic Ocean by large juvenile loggerhead turtles. Roura-Pascual N, editor. Divers Distrib. 2016;22:481–92. https://doi.org/10.1111/ddi.12413
